# Rapid and accurate remethylation of DNA in *Dnmt3a-*deficient hematopoietic cells with restoration of DNMT3A activity

**DOI:** 10.1126/sciadv.adk8598

**Published:** 2024-01-31

**Authors:** Yang Li, Haley J. Abel, Michelle Cai, Taylor A. LaValle, Tiankai Yin, Nichole M. Helton, Amanda M. Smith, Christopher A. Miller, Timothy J. Ley

**Affiliations:** Section of Stem Cell Biology, Division of Oncology, Department of Medicine, Washington University School of Medicine, Saint Louis, MO 63110, USA.

## Abstract

Here, we characterize the DNA methylation phenotypes of bone marrow cells from mice with hematopoietic deficiency of *Dnmt3a* or *Dnmt3b* (or both enzymes) or expressing the dominant-negative *Dnmt3a*^R878H^ mutation [R882H in humans; the most common *DNMT3A* mutation found in acute myeloid leukemia (AML)]. Using these cells as substrates, we defined DNA remethylation after overexpressing wild-type (WT) DNMT3A1, DNMT3B1, DNMT3B3 (an inactive splice isoform of DNMT3B), or DNMT3L (a catalytically inactive “chaperone” for DNMT3A and DNMT3B in early embryogenesis). Overexpression of *DNMT3A* for 2 weeks reverses the hypomethylation phenotype of Dnmt3a-deficient cells or cells expressing the R878H mutation. Overexpression of DNMT3L (which is minimally expressed in AML cells) also corrects the hypomethylation phenotype of *Dnmt3a*^R878H/+^ marrow, probably by augmenting the activity of WT DNMT3A encoded by the residual WT allele. *DNMT3L* reactivation may represent a previously unidentified approach for restoring DNMT3A activity in hematopoietic cells with reduced DNMT3A function.

## Teaser

Hypomethylated regions in Dnmt3a-deficient hematopoietic cells can be restored by short-term overexpression of DNMT3A or DNMT3L.

## INTRODUCTION

DNMT3A and DNMT3B are the dominant mammalian de novo DNA methyltransferases, required for generating CpG methylation patterns in early embryogenesis, and are essential for tissue specification and development (*1*–*4*). In early embryogenesis, the methyltransferase activity of these proteins is greatly augmented by DNMT3L, a highly related chaperone protein that has no DNA methyltransferase activity per se (*5*); however, it forms heterotetramers with DNMT3A or DNMT3B that greatly increase their DNA methyltransferase activities (*6*, *7*). The DNMT3L gene is silenced by epigenetic mechanisms in adult tissues and is minimally expressed in normal or malignant hematopoietic cells (*8*, *9*). However, studies in tissue culture cells have suggested that DNMT3L can be reactivated by hypomethylating agents (HMAs; like 5-azacytidine) and/or histone deacetylase (HDAC) inhibitors, which can synergize to augment its expression (*10*–*13*). Further, when DNMT3L is coexpressed with DNMT3A (but not DNMT3B) in human 293 tissue culture cells, it greatly increases the activity of DNMT3A, increasing methylation at many sites throughout the genome (*14*).

In normal mouse and human hematopoietic cells, the active isoforms of DNMT3A (*DNMT3A1* and *DNMT3A2*) are highly expressed during early development and then gradually silenced during terminal differentiation (*15*). *Dnmt3a* is not absolutely required for hematopoiesis in mice: A complete loss of *Dnmt3a* in the germline yields a phenotypically normal hematopoietic compartment that can be harvested at 2 to 3 weeks of age and transplanted into secondary recipients (*16*). These bone marrow cells have a focal, canonical DNA hypomethylation phenotype, but they can reconstitute all hematopoietic lineages in lethally irradiated recipients (*16*). Hematopoietic stem cells completely deficient for *Dnmt3a* appear to be immortalized with serial transplantation (*17*), and, over time, their progeny develop myeloid skewing (*16*–*19*) and an increased risk of the development of hematopoietic malignancies (*17*–*20*).

Likewise, *Dnmt3b-*deficient bone marrow cells can reconstitute long-term hematopoiesis, but they have reduced B cells, mimicking what is seen in human patients with the “immunodeficiency, centromeric instability, and facial dysmorphism syndrome, type I” [Online Mendelian Inheritance in Man (OMIM), no. 242860] (*1*, *21*, *22*), often caused by loss-of-function mutations in the *DNMT3B* gene. In adult hematopoietic cells and acute myeloid leukemia (AML) cells, the *DNMT3B* gene is expressed, but the dominant isoform is *DNMT3B3*, which does not contain part of the DNA methyltransferase domain (*8*, *9*, *23*). Although DNMT3B3 is inactive as a methyltransferase, recent studies have demonstrated its ability to interact with DNMT3A to augment its activity (*24*–*26*), suggesting that it may have a “chaperone” function for DNMT3A in adult hematopoietic cells, akin to that of DNMT3L in embryonic cells. The DNA methylation phenotype in *Dnmt3b-*deficient bone marrow cells was inferred in a study by Challen *et al.* (*23*), who measured methylation phenotypes in hematopoietic stem/progenitor cells (HSPCs) from *Dnmt3a* or *Dnmt3a × Dnmt3b*-deficient mice, but not from *Dnmt3b*-deficient mice per se. This study suggested that the two enzymes may have unique methylation target sites in the genome, but this has not yet been directly verified in humans or in mice.

While *DNMT3B* mutations are rarely detected in hematopoietic malignancies (*27*–*29*), *DNMT3A* mutations are very common in patients with clonal hematopoiesis and/or myeloid malignancies, and also cause the DNMT3A overgrowth syndrome (DOS). Nearly all *DNMT3A* mutations in these diseases appear to cause loss-of-function, resulting in focal, canonical DNA hypomethylation phenotypes that vary in severity (*18*, *30*–*32*). Deletions and premature stop codons (i.e., causing haploinsufficiency) can cause mild hypomethylation phenotypes that are very similar to that of many missense mutations, suggesting that many of these also cause loss of function (*18*, *33*). Mutations at amino acid R882 in humans (R878 in mice) cause a more severe hypomethylation phenotype, probably reflecting the dominant-negative activity of these mutations (*8*, *18*); they directly reduce methyltransferase activity by ~80%, and the mutant protein also preferentially interacts with wild-type (WT) DNMT3A, trapping it in inactive heterodimeric complexes (*8*). Despite the methylation consequences of these mutations, patients with DOS and patients with clonal hematopoiesis can live for many years (or even decades) with essentially normal blood counts, so the effects on blood cell development are relatively small and slow to develop. Because nearly all *DNMT3A* mutations are heterozygous, a residual WT allele is usually present and expressed, providing a potential source of WT DNMT3A that could potentially overcome the loss-of-function or dominant-negative effects of mutations, if its activity could be increased.

In this study, we use whole-genome bisulfite sequencing (WGBS) to define the DNA methylation phenotypes of the bone marrow cells of adult mice that had undergone somatic inactivation of *Dnmt3a* or *Dnmt3b* (or both enzymes) at embryonic days 9 to 10, using *Vav1-Cre* to induce near-complete floxing of these alleles in hematopoietic cells (*34*, *35*). Likewise, we define the methylation phenotype of *Dnmt3a*^R878H/+^ × *Vav1-Cre* bone marrow cells, which is intermediate in severity between haploinsufficiency and complete loss of *Dnmt3a* expression. In all these studies, bone marrow cells were derived directly from unmanipulated mice, eliminating potential methylation phenotypes caused by the stress of transplantation (which increases HSPC cycling and the expression of *Dnmt3a*) (*9*). We have previously shown that the differentially methylated regions (DMRs) in *Dnmt3a*-deficient HSPCs, progenitor populations, mature B cells, and mature myeloid cells are nearly identical (*16*), strongly suggesting that Dnmt3a must perform de novo methylation predominantly in early stem/progenitor cells; this methylation phenotype is probably maintained in differentiated progeny by Dnmt1 (*15*, *16*). These observations make it possible to examine the consequences of *Dnmt3a* mutations in unfractionated whole–bone marrow samples, because all of the purified populations have similar methylation phenotypes at regions where DNMT3A acts.

Last, we explore the effects of retroviral overexpression of *DNMT3A1*, *DNMT3B1*, *DNMT3B3*, and *DNMTL* on the DNA methylation phenotypes of bone marrow cells deficient for Dnmt3a, Dnmt3b, or both enzymes. Hypomethylation phenotypes were corrected within weeks by overexpressing DNMT3A1 or DNMT3B1. Methylation can also be completely and rapidly corrected in cells expressing a heterozygous *Dnmt3a*^R878H/+^ mutation by overexpressing *DNMT3L*, which may act to increase the activity of WT DNMT3A protein encoded by the residual WT allele. These data clarify the contributions of DNMT3A and DNMT3B to the methylation patterns of adult hematopoietic cells and suggest a potential strategy for increasing the activity of DNMT3A in AML cells initiated by *DNMT3A* mutations.

## RESULTS

### Focal, canonical hypomethylation phenotypes in the bone marrow cells of *Dnmt3a-* or *Dnmt3b*-deficient mice

**Fig. 1.**
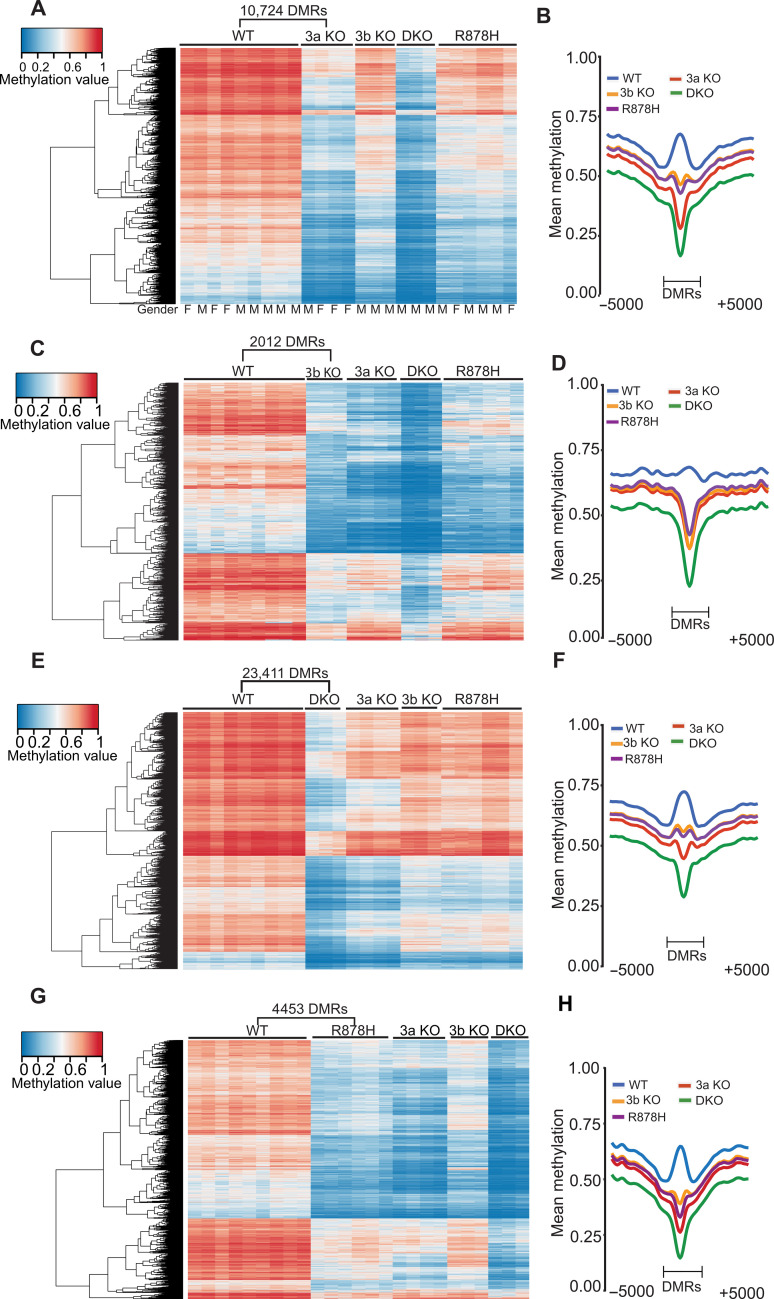
DNA methylation phenotypes of *Dnmt3a*, *Dnmt3b*, or doubly deficient mouse bone marrow cells. (**A**) Heatmap showing the methylation values for 10,724 DMRs defined by comparing WGBS data from wild-type (WT, *n* = 9) versus *Dnmt3a^flox/flox^ × Vav1-Cre* (3a KO, *n* = 4) samples. Values for the same DMRs were plotted passively for *Dnmt3b^flox/flox^ × Vav1-Cre* (3b KO), *Dnmt3a^flox/flox^ × Dnmt3b^flox/flox^ × Vav1-Cre* (DKO), and *Dnmt3a^R878H/+^* × *Vav1-Cre* (R878H) samples. (**B**) Aggregate (mean) methylation values from the 10,724 DMRs identified in (A) for each genotype. (**C**) Heatmap showing the methylation values of 2012 DMRs defined by comparing WGBS data from WT (*n* = 9) versus 3b KO (*n* = 3) samples. Values for the same DMRs were plotted passively for the 3a KO, 3b KO, DKO, and R878H samples. (**D**) Aggregate (mean) methylation values from the 2012 DMRs identified in (C) for each genotype. (**E**) Heatmap showing the methylation values of 23,411 DMRs defined by comparing WGBS data from WT (*n* = 9) versus DKO (*n* = 3) samples. Values for the same DMRs were plotted passively for the 3a KO, 3b KO, and R878H samples. (**F**) Aggregate (mean) methylation values from the 23,411 DMRs identified in (E) for each genotype. (**G**) Heatmap showing the methylation values of 4453 DMRs defined by comparing WGBS data from WT (*n* = 9) versus R878H (*n* = 6) samples. Values for the same DMRs were plotted passively for *the* 3a KO, 3b KO, and DKO samples. (**H**) Aggregate (mean) methylation values from the 4453 DMRs identified in (G) for each genotype.

Previous studies of the methylation patterns of *Dnmt3a*-deficient hematopoietic cells have predominantly used transplanted cells from mice with germline mutations (*16*) or conditional knockout mice that have been serially transplanted (*17*). To better understand the consequences of the loss of DNA methyltransferases in unmanipulated hematopoietic cells, we used the *Vav1-Cre* transgene to induce floxing at embryonic days 9 to 10 in mice with *Dnmt3a^flox/flox^*, *Dnmt3b^flox/flox^*, or *Dnmt3a^flox/flox^* × *Dnmt3b^flox/flox^* alleles. We determined floxing efficiency in the bone marrow samples of every tested mouse using sequence coverage of the floxed (i.e., deleted) regions of each gene, measured in the whole-genome bisulfite data (fig. S1A); the mean value for all mice was 96%. Mice from these crosses were born at the expected Mendelian frequencies and had no overt abnormalities in growth or development. At 6 to 8 weeks of age, their blood counts were not significantly different from those in WT mice (fig. S1B). Whole–bone marrow cells were harvested from mice at 6 to 12 weeks of age for DNA methylation studies. WGBS was performed on all samples to define the CpG methylation phenotypes across the genome. For all WGBS samples combined in all studies, we achieved a mean genome coverage of 18.6× (minimum coverage, 13.1×). We first defined DMRs by comparing four bone marrow samples from *Dnmt3a^flox/flox^ × Vav1-Cre* mice (henceforth “3a KO,” one male and three females) to nine WT bone marrow samples (“WT,” six males and three females) using previously published methods, to identity *Dnmt3a*-dependent methylation changes (Fig. 1, A and B). DMRs were defined as having at least 10 CpGs, a mean methylation difference between two groups of ≥0.2 and a false discovery rate (FDR) of <0.05. Contiguous DMRs within 50 base pairs (bp) of each other were combined. We identified 10,724 DMRs (Fig. 1A and table S1) in the 3a KO mice; there were no differences based on the sex of the mice. The average width of these DMRs was 746 bp, and the mean number of CpGs per DMR was 19.5. In total, these DMRs encompassed ~8 Mb of DNA, accounting for about 0.3% of the mouse genome. A total of 10,714 of the 10,724 DMRs (~99.9%) were hypomethylated in the 3a KO bone marrow samples. We also “passively” plotted the mean methylation values for the same DMRs in *Dnmt3b*^flox/flox^ × *Vav1-cre* bone marrow samples (“3b KO”), samples from mice deficient for both enzymes (“DKO”), or samples from *Dnmt3a*^R878H/+^ × *Vav1-Cre* mice (“R878H”). The 3b KO and R878H samples had more subtle methylation changes at these 10,724 DMRs, and the DKO samples had more pronounced reductions in methylation. The aggregated mean methylation values for the 3a KO DMRs are shown in a summary “canyon plot” in Fig. 1B, which quantifies the average differences in methylation for each genotype at the 3a KO DMRs.

A comparison of the same nine WT samples and three 3b KO samples (all males) yielded 2012 DMRs (Fig. 1, C and D), with a mean size of 597 bp and an average of 17 CpGs per DMR, encompassing ~1.2 Mb (0.045% of the mouse genome). A total of 2009 of the 2012 DMRs (~99.9%) were hypomethylated. Passive plotting of the mean methylation values of the 3b KO DMRs was performed for the 3a KO samples, the DKO samples, and the R878H samples (Fig. 1C and table S2). In most of these regions, the 3a KO samples were likewise hypomethylated, suggesting that Dnmt3a and Dnmt3b may act at many of the same genomic regions. However, some regions in the 3b KO samples were more hypomethylated than the 3a KOs; manual review of these regions revealed that the vast majority also had reduced methylation in the 3a KO samples (only two DMRs appeared to be 3b KO specific). The mean methylation values of 3b KO DMRs were further reduced in the DKO samples, again suggesting that the DNMT3A and DNMT3B may be acting synergistically at many sites. The R878H methylation values at 3b KO DMRs were similar to those of the 3a KO samples. Comparative quantification of the aggregated mean methylation values of these DMRs for all genotypes is shown in the canyon plot and is consistent with the observations in the heatmap (Fig. 1D).

We next defined DMRs in the DKO bone marrow samples (three males) versus the same nine WT samples. We detected 23,411 DMRs (Fig. 1, E and F), of which 23,408 of the 23,411 DMRs (99.99%) were hypomethylated. These DMRs span 21.4 Mb of DNA in total (~0.8% of the mouse genome). The mean size of these DMRs was 916 bp, with an average of 23 CpGs per DMR. Passive plotting of these DMRs with data from the other genotypes revealed that the DKO DMRs were the most hypomethylated and that all genotypes have reduced DNA methylation at these sites in the genome (Fig. 1F and table S3).

We also evaluated the phenotypes of *Dnmt3a^R878H/+^* × *Vav1-Cre* mice (using the floxed allele established by (*36*). Using RNA sequencing (RNA-seq) data from the bone marrows of WT and R878H mice, we determined that the mean floxing efficiency in these mice was >96%. These mice had no gross phenotypic abnormalities at 6 to 12 weeks of age (i.e., no overgrowth or obesity) (*15*) and had essentially normal complete blood counts (fig. S1B). We harvested bone marrow cells from six *Dnmt3a^R878H/+^* mice (R878H, four males and two females) for WGBS. By comparing these data to WT bone marrow samples, we identified 4453 DMRs in the R878H samples (Fig. 1, G and H, and table S4), of which 4450 were hypomethylated (99.9%). The average size of R878H DMRs was 672 bp, and each contained a mean of 20 CpGs; these DMRs encompassed ~3 Mb of DNA (~0.1% of the mouse genome). Passive plotting of these DMRs for the other genotypes revealed that all had some reduction of methylation at virtually all sites; this reduction was uniformly more pronounced for the 3a KO and DKO samples. No differences could be ascribed to the sex of the animals.

**Fig. 2.**
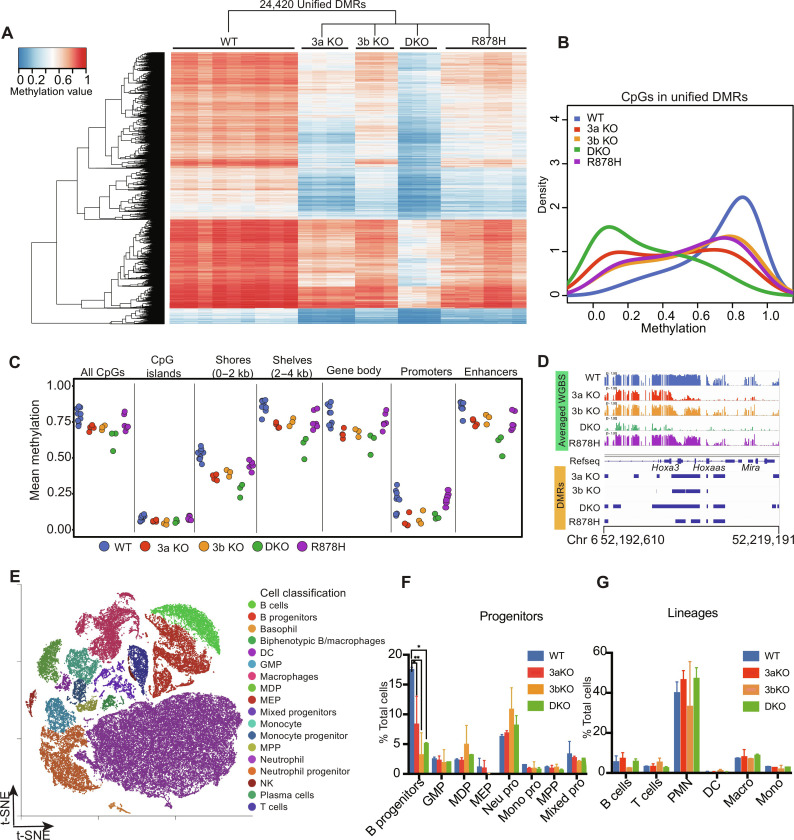
Characterization of methylation and cellular phenotypes in DNA methyltransferase–deficient mouse bone marrow cells. (**A**) Heatmap showing the methylation values for individual samples of DMRs from combining all the DMRs (“Unified” DMRs) generated from the comparisons of WT versus 3a KO, WT versus 3b KO, WT versus DKO, and WT versus R878H samples. (**B**) Density plot of mean methylation values from the unified DMRs for 3a KO, 3b KO, DKO, and R878H samples*.* (**C**) Mean CpG methylation values from WGBS of bone marrow cells from WT, *3a* KO, 3b KO, DKO, and R878H samples. Mean values across all CpGs in annotated regions of the genome are shown. (**D**) IGV view of a representative region of the *Hoxa* gene cluster. Mean methylation values for each CpG are shown as a bar ranging from 0 to 100% methylated for each genotype. Blue bars at the bottom indicate DMRs identified in this region. (**E**) t-distributed Stochastic Neighbor Embedding (t-SNE) projections of merged scRNA-seq data from whole–bone marrow cells derived from WT (*n* = 3, two males and one female), 3a KO, 3b KO, and DKO mice, 6 to 8 weeks of age (*n* = 2 for each for each knockout genotype; all samples were from males) Cell populations were assigned according to the Haemopedia algorithm and manual review. B progenitors include pro-B cells and pre-B cells; DC, dendritic cell; GMP, granulocyte-monocyte progenitors; MDP, monocyte dendritic cell progenitors; MEP, megakaryocyte erythrocyte progenitor; MPP, multipotent progenitor; NK, natural killer cells. (**F**) Progenitor population distributions for each genotype using the scRNA-seq data in (E). *P < 0.05 and **P < 0.01 via analysis of variance (ANOVA) test. (**G**) Mature cell population distributions for each genotype, using the scRNA-seq data in (E). PMN, polymorphonuclear leukocyte, including neutrophils and basophils in (E); Macro, macrophages; Mono, monocytes.

To evaluate the global methylation defects caused by loss of function of Dnmt3a and Dnmt3b, we took the union of all DMRs from the above comparisons, merging overlaps to create a “unified set” of 24,420 DMRs, plotted for all genotypes in Fig. 2A (table S5). No consistent differences could be ascribed to the sexes of the mice. As expected, DKO samples had the lowest methylation levels at nearly all DMRs. 3a KO methylation values were the next most hypomethylated, suggesting the important role of *Dnmt3a* in defining these DMRs. To extend these findings to all CpGs (i.e., not just DMRs), we first performed a global analysis of CpG methylation values for all samples in the methylation density plot shown in Fig. 2B. The relative methylation values for all CpGs were WT > 3b KO ≥ R878H > 3a KO > DKO. A plot showing CpG methylation values in annotated regions of the genome for all samples is shown in Fig. 2C (also see fig. S2, A and B); because DMRs encompass less than 1% of the genome, the overall differences from WT samples were relatively small. All of the differences from WT samples were statistically significant. The most notable changes in methylation were found in shores, shelves, gene bodies, and enhancers, which are much more methylated than CpG islands and promoters. In all regions, the DKO samples had the most notable reductions in methylation, and the R878H samples tended to have milder changes. To directly visualize the differential methylation phenotypes for all genotypes at a typical, informative DMR (in the *HoxA* gene cluster), we show an Integrative Genomics Viewer (IGV) view of the methylation values for all CpGs in this region in Fig. 2D. The size and shape of the DMRs in this region reflect the depth and width of DMRs genome-wide for these genotypes, providing context for the average values shown for methylation in the heatmaps. An IGV view of each sample plotted individually for this locus is shown in fig. S2C (highlighted by red box), demonstrating the reproducibility of this phenotype across many WGBS samples from different mice.

Last, to confirm that the altered methylation phenotypes are due to the cell-intrinsic loss of DNA methyltransferases per se (and not to shifts in hematopoietic cell populations with altered DNA methylation patterns) (*15*), we used the 10X Genomics Chromium platform to perform single-cell RNA-seq (scRNA-seq) on whole–bone marrow cells derived from two independent mice each (6 to 12 weeks of age) for WT, 3a KO, 3b KO, and DKO mice. Cell identities were inferred on the basis of the Haemopedia Database (*37*) and graphic-based manual review (Fig. 2E and fig. S2D). The numbers of early B cells were significantly reduced in 3a KO, 3b KO, and DKO marrows compared to those in WT, with 3b KOs showing the most reduction (Fig. 2F). The mature B cell population in 3b KO cells was also reduced, but the change was not statistically significant (Fig. 2G). The sizes of all other populations were not statistically different from WT marrow samples. Collectively, these data suggest that the genotype-specific change in DNA methylation is not due to markedly altered marrow populations but rather due to the loss-of-function mutations themselves.

### Correction of *Dnmt3a* KO DMRs by addback of DNA methyltransferases

**Fig. 3.**
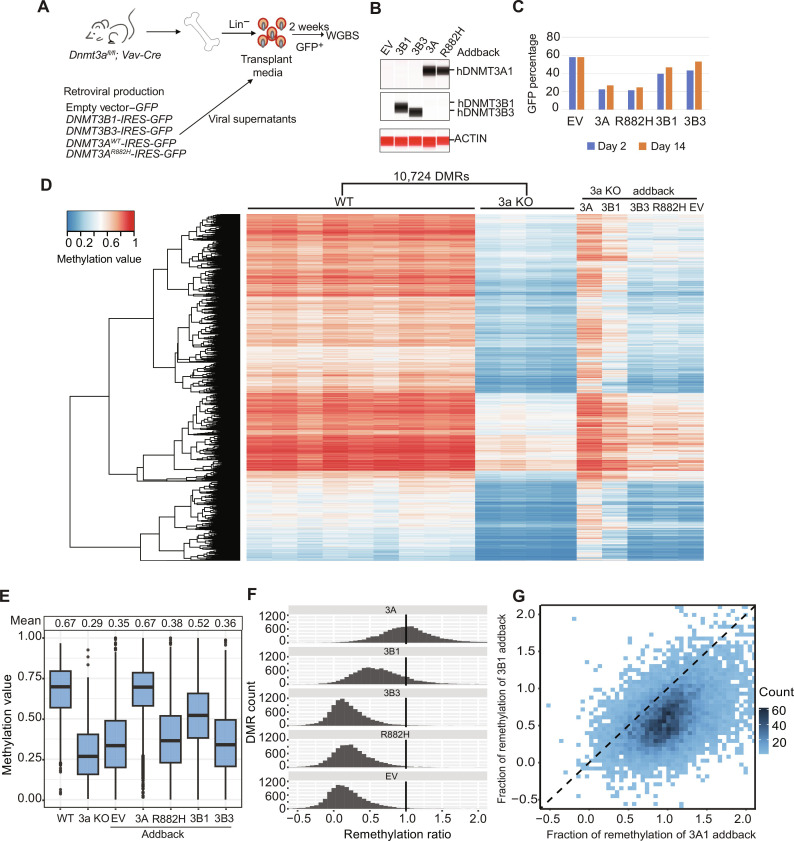
Effects of retroviral addback ex vivo in primary 3a KO bone marrow cells. (**A**) Schematic workflow for an ex vivo addback experiment using *Dnmt3a^−/−^* primary mouse bone marrow cells. (**B**) ProteinSimple “Western blot” showing DNA methyltransferase protein abundance in *Dnmt3a^−/−^* lineage depleted bone marrow cells, 2 days after transduction. (**C**) Fraction of transduced (i.e., GFP^+^) *Dnmt3a^−/−^* bone marrow cells at 2 and 14 days after transduction. (**D**) Heatmap showing the methylation values for 10,724 DMRs (from Fig. 1A) defined by comparing WGBS data from WT (*n* = 9) versus 3a KO (*n* = 4) samples. DNA methylation values for the same DMRs were plotted passively for purified GFP^+^ cells from the indicated retroviral vectors into *Dnmt3a^−/−^* bone marrow progenitors, after 14 days in liquid culture. “3A,” *DNMT3A1* cDNA–expressing vector; “3B1,” *DNMT3B1* cDNA vector; “3B3,” *DNMT3B3* cDNA vector; “R882H,” *DNMT3A1*^R882H^ cDNA vector. EV, empty vector (i.e., no cDNA inserted). (**E**) Mean methylation values for all of the 3a KO DMRs in WT, 3a KO, and transduced 3a KO samples from the WGBS data shown in (D). (**F**) Extent of methylation correction (i.e., the methylation difference between addback and 3a KO scaled by the methylation difference between WT and 3a KO) for all the 3a KO DMRs in non-transduced or transduced 3a KO samples. The vertical line at 1.0 indicates remethylation of DMRs to WT levels. (**G**) Extent of remethylation of *Dnmt3a^−/−^* DMRs in *DNMT3A1*-transduced 3a KO bone marrow cells versus *DNMT3B1*-transduced *Dnmt3a^−/−^* bone marrow cells.

To determine whether the hypomethylation phenotype of *Dnmt3a-*deficient mouse bone marrow could be corrected with a genetic addback approach, we created retroviral vectors to express full-length human *DNMT3A1*, *DNMT3B1*, or *DNMT3B3 cDNAs* in mouse hematopoietic cells. We used human cDNAs because these genes are highly conserved between mice and humans (fig. S3A) and because we have previously shown that a human *DNMT3A1* transgene can completely correct the focal hypomethylation phenotype in *Dnmt3a*-deficient mouse hematopoietic cells (*14*); further, our long-term goal is to use proven cDNAs to restore DNMT3A function in human cells. To define the most common splicing isoforms expressed in mouse bone marrow cells, we analyzed bulk RNA-seq data from WT versus R878H mice, which revealed that the dominant isoform of *Dnmt3a* is *Dnmt3a1* [88.6% of transcripts in WT (*n* = 10; range, 63 to 100%) and 88.5% in R878H (*n* = 12; range, 73 to 100%), *P* = 1.0], and the dominant isoform of *Dnmt3b* is *Dnmt3b3* [78.2% of transcripts in WT (*n* = 10; range, 63 to 100%) and 78.1% in R878H (*n* = 12; range, 37 to 100%), *P* = 1.0] (Fig. 3A and fig. S3, B and C). Transduced cells were harvested 2 days after back-to-back daily transductions to define the fractions of cells that were green fluorescent protein (GFP)^+^; purified GFP^+^ cells were used to perform quantitative Western blots (using the “ProteinSimple” platform) to ascertain the level of expression of proteins from each transduced retrovirus; no endogenous DNMT3A was detected in the 3a KO cells, but this protein was expressed in cells transduced either with WT *DNMT3A1* or the same cDNA with the R882H mutation (which reduces methyltransferase activity by ~80%) (Fig. 3, B and C); compared to endogenous DNMT3B3 levels detected in the empty vector (EV)–transduced cells on same Western blot (Fig. 3B), retroviral DNMT3B1 levels were increased ~17-fold, and DNMT3B3 levels were increased ~16-fold. Transduced cells were then placed in “transplant media” [RPMI 1640 with 15% fetal bovine serum (FBS) and murine interleukin-3 (IL-3), stem cell factor (SCF), FMS-like tyrosine kinase 3 ligand (FLT3L), and thrombopoietin (TPO)] for 2 weeks, and GFP percentages were determined; fractions of GFP^+^ cells were minimally altered from baseline values (Fig. 3C), suggesting that overexpression of these proteins does not select for or against expressing cells over this time frame. DNA methylation was assessed by performing WGBS from DNA harvested from purified GFP^+^ cells at day 14 and is displayed in heatmap form in Fig. 3D. DMRs previously defined for WT versus 3a KO bone marrow samples were used to calibrate DNA methylation changes caused by Dnmt3a deficiency, and methylation values for each of the “addback” samples were plotted passively at these genomic locations. *DNMT3A1* addback to 3a KO samples remethylated nearly all of the 3a KO DMRs. Overexpression of *DNMT3B1* partially remethylated the 3a KO DMRs. Overexpression of *DNMT3B3*, *DNMT3A^R882H^*, and the MSCV “EV” minimally altered methylation of the 3a KO DMRs, as expected. The data presented in the heatmap are quantified (Fig. 3, E to G) to reveal that nearly all of the hypomethylated 3a DMRs are remethylated by DNMT3A1 overexpression for 2 weeks, without evidence for excess methylation at these sites. In contrast, *DNMT3B1* overexpression remethylated a large fraction of the 3a KO DMRs but less efficiently than *DNMT3A1* (Fig. 3, F and G). Overexpression of *DNMT3B3* or *DNMT3A*^R882H^ had minimal effects on remethylation, similar to that of the empty retroviral vector.

### DNMT3B3 facilitates DNA remethylation in a DNMT3A-dependent manner

**Fig. 4.**
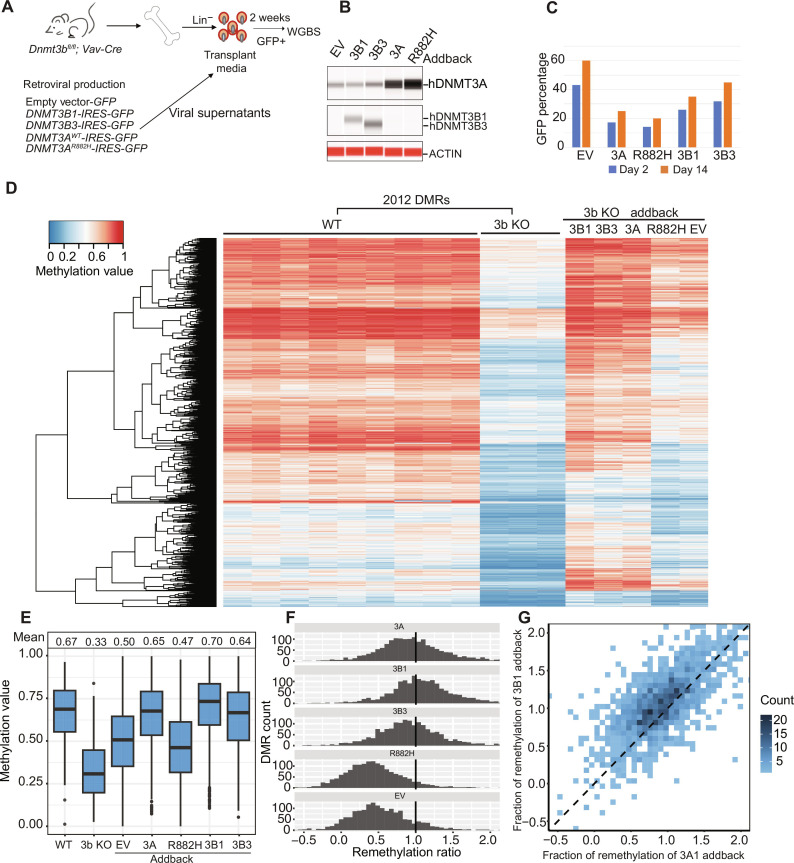
Effects of retroviral addback ex vivo in primary 3b KO bone marrow cells. (**A**) Schematic workflow for an ex vivo addback experiment using 3b KO primary mouse bone marrow cells. (**B**) ProteinSimple Western blot showing DNA methyltransferase protein abundance in 3b KO lineage depleted bone marrow cells, 2 days after transduction. (**C**) Percentage of transduced, GFP^+^ 3b KO bone marrow cells at 2 and 14 days after transduction. (**D**) Heatmap showing the methylation values for 2012 DMRs (from Fig. 1B) defined by comparing WGBS data from WT (*n* = 9) versus 3b KO (*n* = 3) samples. DNA methylation values for the same DMRs were plotted passively for purified GFP^+^ cells from the indicated retroviral vectors into 3b KO bone marrow progenitors, after 14 days in liquid culture. (**E**) Mean methylation values for all the 3b KO DMRs in *WT*, 3b KO, and transduced 3b KO samples from the WGBS data shown in (D). (**F**) Extent of correction of methylation values for all the 3b KO DMRs in non-transduced or transduced 3b KO samples. The vertical line drawn at 1.0 indicates the level of methylation for these DMRs in WT bone marrow cells. (**G**) Extent of remethylation of 3b KO DMRs in *DNMT3A1*-transduced 3b KO bone marrow cells, versus *DNMT3B1*-transduced 3b KO bone marrow cells.

RNA-seq analysis of adult mouse bone marrow cells revealed that the majority of *Dnmt3b* transcripts are of the *Dnmt3b3* isoform, which does not contain a large portion of the methyltransferase domain (fig. S3C). To further define the functions of *DNMT3B1* and *DNMT3B3* in hematopoietic cells, we did an ex vivo addback experiment using *Dnmt3b*-deficient mouse bone marrow cells, using an experimental strategy identical to the one shown in Fig. 3 (Fig. 4A). We confirmed overexpression of appropriately sized DNMT3B proteins by Western blotting of samples obtained 2 days after transduction (Fig. 4B); in these samples, we could define the endogenous level DNMT3A protein expression and determined that WT DNMT3A1 was overexpressed 2.5-fold compared to the level detected in EV-transduced cells and that DNMT3A1^R882H^ was overexpressed ~3.7-fold (the smaller apparent fold change for DNMT3A [~3× versus ~16× for 3B] probably reflects the 5.4-fold higher background level of endogenous *Dnmt3a* mRNA in mouse bone marrow cells, which reduces the apparent fold change that can be achieved for 3A; fig. S3D). Flow cytometry revealed that the fraction of GFP^+^ cells was similar for all constructs at days 2 and 14 (Fig. 4C). DNA was harvested from GFP^+^ cells for all transductions on day 14 and subjected to WGBS (Fig. 4, D to G). Methylation values of DMRs defined by comparing WT versus 3b KO bone marrow cells (*n* = 2012) were used to define the impact of the addbacks, which were passively plotted for the same DMRs. As expected, *DNMT3B1* restored the methylation values of the 3b KO DMRs to levels that were nearly equivalent to WT samples (Fig. 4, D to F). Likewise, DNMT3A1 restored methylation at the 3b KO DMRs to levels that were nearly equivalent to WT or DNMT3B1 addback cells, suggesting that DNMT3A1 and DNMT3B1 can remethylate the same regions of the genome (Fig. 4G). *DNMT3B3* overexpression likewise induced remethylation of the 3b KO DMRs to near WT levels, suggesting that DNMT3B3 may be augmenting the activity of DNMT3A to cause remethylation at these sites; DNMT3B3 was shown to be completely inactive in addback experiments with 3a KO cells (see above) or DKO cells (see below). We also noted that the EV and *DNMT3A*^R882H^ addback caused some remethylation at the 3b KO DMRs, suggesting that the proliferative stress of tissue culture expansion under the influence of four potent hematopoietic cytokines (IL-3, TPO, FLT3L, and SCF) increases the expression of endogenous *Dnmt3a* (*9*). We demonstrated that DNMT3A protein levels are high in unmanipulated, lineage negative, WT bone marrow cells (due to progenitor enrichment, where *Dnmt3a* is expressed at its highest level) and that high levels of DNMT3A persist for 14 days in this culture system, which contains high levels of hematopoietic growth factors that cause proliferative stress (fig. S4).

**Fig. 5.**
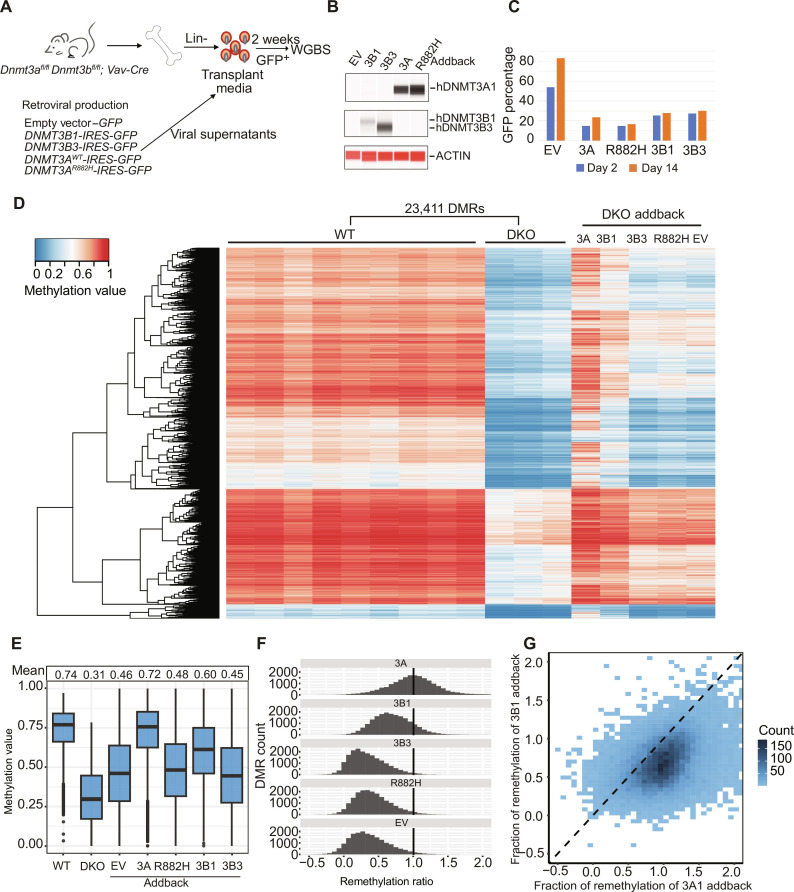
Effects of retroviral addback ex vivo in primary DKO bone marrow cells. (**A**) Schematic workflow for an ex vivo addback experiment using DKO primary mouse bone marrow cells. (**B**) ProteinSimple Western blot showing DNA methyltransferase protein abundance in DKO lineage depleted bone marrow cells, 2 days after transduction. (**C**) Percentage of transduced, GFP^+^ DKO bone marrow cells at 2 and 14 days after transduction. (**D**) Heatmap showing the methylation values for 23,411 DMRs (from Fig. 1E) defined by comparing WGBS data from WT (*n* = 9) versus DKO (*n* = 3) samples. DNA methylation values for the same DMRs were plotted passively for purified GFP^+^ cells from the indicated retroviral vectors into DKO bone marrow progenitors, after 14 days in liquid culture. (**E**) Mean methylation values for all the *DKO* DMRs in *WT*, DKO, and transduced DKO samples from the WGBS data shown in (D). (**F**) Extent of correction of methylation values for all the DKO DMRs in non-transduced or transduced DKO samples. The vertical line drawn at 1.0 indicates the level of methylation for these DMRs in WT bone marrow cells. (**G**) Extent of remethylation of DKO DMRs in *DNMT3A1*-transduced DKO bone marrow cells versus *DNMT3B1*-transduced DKO bone marrow cells.

To extend these findings, we used the same experimental strategy to perform addback experiments using DKO mouse bone marrow cells (Fig. 5A). Expression of each protein in GFP^+^ cells on day 2 was confirmed by ProteinSimple Western assays (Fig. 5B); the fraction of GFP^+^ cells in each transduction was similar at days 2 and 14 (Fig. 5C). The methylation values of DKO versus WT DMRs (*n* = 23,411) were used to calibrate the heatmap showing in Fig. 5D, and methylation values for the addback samples were plotted passively. DNMT3A1 addback restored methylation values at these DMRs to near WT levels (Fig. 5, E and F). DNMT3B1 addback partially remethylated these sites, but not as efficiently as DNMT3A1 (Fig. 5G); however, we cannot definitively determine whether levels of DNMT3A1 and DNMT3B1 overexpression were equivalent because unique antibodies (presumably with different affinities for their target proteins) were used to detect their expression. As expected, the EV, *DNMT3B3-*, and *DNMT3A*^R882H^-expressing vectors had equivalent levels of methylation at DKO DMRs. Some “remethylation” of DKO DMRs was detected, which may reflect normal DNA methylation in the small fraction of residual WT cells (i.e., non-floxed) in these samples.

In summary, the addback experiments described above suggest that DNMT3A is the dominant de novo DNA methyltransferase in hematopoietic cells and that DNMT3B3 primarily acts as a chaperone to augment the activity of DNMT3A, consistent with the observations of Duymich *et al*. (*25*) and Zeng *et al*. (*26*). These data also suggested to us that DNMT3L, which is minimally expressed in both human and mouse hematopoietic cells (*7*, *38*), might also be capable of augmenting the activity of DNMT3A with addback. In the next set of experiments, we tested the ability of overexpressed DNMT3A and DNMT3L to overcome the dominant-negative effect of the heterozygous *Dnmt3a*^R878H^ mutation in hematopoietic cells.

### DNMT3L potentiates DNMT3A activity more than DNMT3B3

Both DNMT3L and DNMT3B3 can facilitate DNA methylation in the presence of DNMT3A (*6*), but the relative abilities of each to augment DNMT3A have not been defined. We designed an experiment to test this question, using recombinant proteins. We transiently transfected human embryonic kidney (HEK) 293T cells with eukaryotic expression plasmids (using a pcDNA3.1 backbone) containing His-tagged *DNMT3A1*, with or without DNMT3L or DNMT3B3 (both without His-tags). Two days after transfection, we purified DNMT3A using immobilized metal affinity chromatography Nickel resin columns, as previously described (*8*). Tryptic peptides from the enriched proteins were identified with mass spectrometry, and protein abundance was defined after normalization to DNMT3A content in each sample and to protein mass (fig. S5A and table S6). When *DNMT3A1* was transfected alone, little or no endogenous DNMT3B or DNMT3L was copurified with the His-tagged DNMT3A. However, when *DNMT3A1* was cotransfected with *DNMT3B3*, a large amount of DNMT3B was copurified (the ratio of DNMT3A:DNMT3B was 1.3:1); similarly, cotransfection of *DNMT3A1* and *DNMT3L* yielded a large amount of copurified DNMT3L (the ratio of DNMT3A:DNMT3L was 1:4:1). Therefore, in this system, the accessory proteins DNMT3B3 or DNMT3L are “captured” and copurified via their interactions the His-tagged DNMT3A1. Methyltransferase activities of the purified proteins from each transfection (performed in triplicate) were determined using an in vitro methyltransferase assay, as previously described (fig. S5B) (*7*). Equivalent amounts of immunoreactive DNMT3A (defined by quantitative Western blotting) were evaluated in each assay. The methyltransferase activity of co-purified DNMT3A and DNMT3B3 was significantly increased compared to that of DNMT3A alone (49% increase). The methyltransferase activity of co-purified of DNMT3A and DNMT3L was significantly increased as well and 5.5 times higher than that of DNMT3A alone. These data strongly suggest that DNMT3L is more active than DNMT3B3 as a DNMT3A1 chaperone in vitro. We also evaluated the ability of DNMT3B3 and DNMT3L to augment the activity of purified recombinant DNMT3A^R882H^ protein, which is much less active than WT DNMT3A (fig. S5B). DNMT3B3 coexpression resulted in a nonsignificant augmentation of activity, but DNMT3L coexpression increased its activity 4.2-fold, similar to that of the 5.5-fold augmentation of WT DNMT3A.

### Overexpression of DNMT3A or DNMT3L can overcome the hypomethylation defect caused by the dominant-negative *Dnmt3a^R878H/+^* mutation in hematopoietic cells

**Fig. 6.**
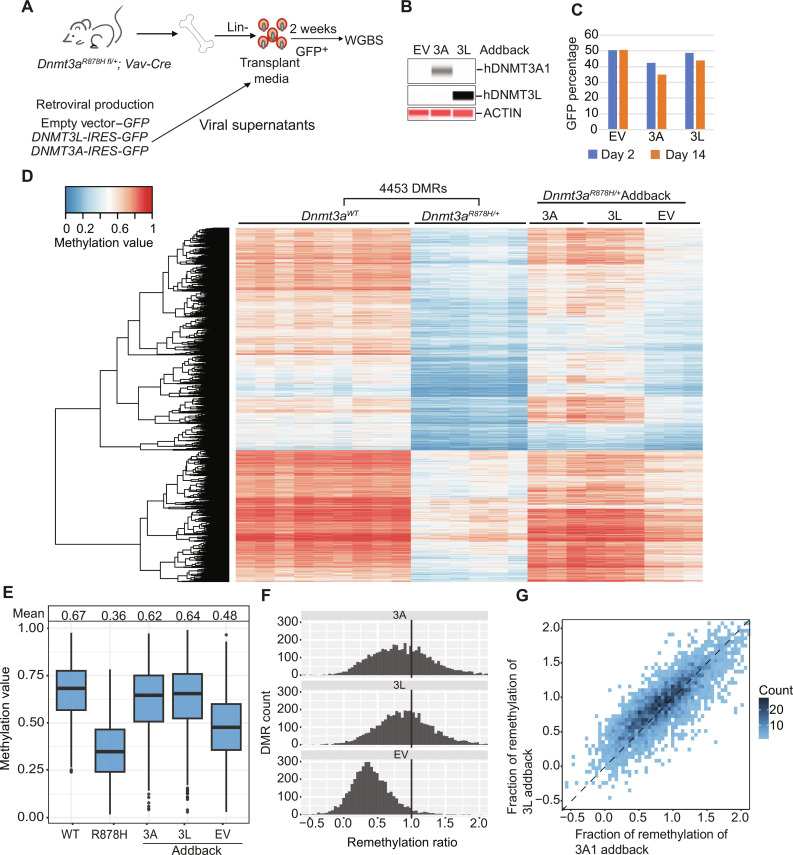
Effects of retroviral addback ex vivo in primary R878H bone marrow cells. (**A**) Schematic workflow for an ex vivo addback experiment using R878H primary mouse bone marrow cells. (**B**) ProteinSimple Western blot showing DNA methyltransferase protein abundance in R878H lineage depleted bone marrow cells, 2 days after transduction. (**C**) Percentage of transduced, GFP^+^ R878H bone marrow cells at 2 and 14 days after transduction. (**D**) Heatmap showing the methylation values for 4453 DMRs defined by comparing WGBS data from WT (*n* = 9) versus R878H (*n* = 6) samples. DNA methylation values for the same DMRs were plotted passively for purified GFP^+^ cells (i.e., transduced) from the indicated retroviral vectors into R878H bone marrow progenitors, after 14 days in liquid culture. “3L,” *DNMT3L.* (**E**) Mean methylation values for all the R878H DMRs in WT, R878H, and transduced R878H samples from the WGBS data shown in (D). (**F**) Extent of correction of methylation values for all the R878H DMRs in non-transduced or transduced R878H samples. The vertical line drawn at 1.0 indicates the level of methylation for these DMRs in WT bone marrow cells. (**G**) Extent of remethylation of R878H DMRs in *DNMT3A1*-transduced R878H bone marrow cells versus *DNMT3L*-transduced R878H bone marrow cells.

To perform these experiments, we used a human DNMT3L cDNA in a retroviral construct. To determine whether human DNMT3L could efficiently interact with mouse DNMT3A, we coexpressed MYC-tagged human *DNMT3L* cDNA with either a mouse *Dnmt3a1* cDNA or a human *DNMT3A1* cDNA (neither of which were MYC-tagged). Pull-down experiments with an anti-MYC antibody followed by Western blotting revealed that both mouse and human DNMT3A1 were capable of interacting with human DNMT3L (fig. S5C). Retroviral addback experiments using an empty MSCV vector or vectors containing *DNMT3A1* or *DNMT3L* cDNAs were transduced into bone marrow cells from of 6- to 8-week-old *Dnmt3a^R878H/+^* x *Vav1-Cre* mice (Fig. 6A). GFP^+^ cells were purified after 2 days of in vitro culture exactly as described above and assessed for overexpression of each protein by Western blotting (Fig. 6B). GFP^+^ cells from both vectors were minimally changed after 14 days of culture, suggesting that these proteins did not select for or against expressing cells (Fig. 6C). GFP^+^ cells were purified after 14 days from three biological replicates, and WGBS was performed. Methylation data for these samples were plotted passively for the DMRs defined for R878H versus WT bone marrow cells (Fig. 6D). Although some remethylation was observed with the EV addback alone (for reasons described above), near-complete remethylation was observed with either DNMT3A1 or DNMT3L overexpression (Fig. 6, E and F); remethylation was virtually indistinguishable with the two vectors (Fig. 6G). The identical experiment, when performed with 3a KO cells as the substrate, revealed near-complete restoration of methylation with *DNMT3A1* addback but no remethylation (over background) with *DNMT3L* overexpression (fig. S6). These data demonstrate that DNMT3L has no intrinsic activity in this system in the absence of DNMT3A, and confirms that remethylation in R878H cells requires an intact WT *Dnmt3a* allele.

### Overexpression of DNMT3A or DNMT3L can overcome the hypomethylation defect caused by the *DNMT3A*^R878H/+^ mutation in vivo

**Fig. 7.**
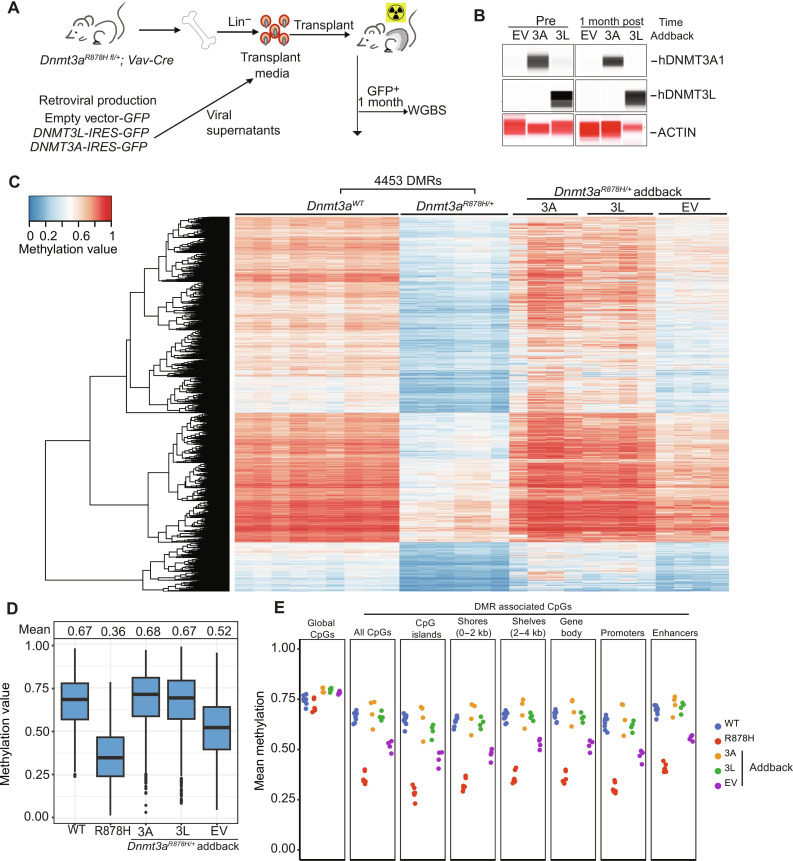
Effects of retroviral addback in vivo with R878H bone marrow cells. (**A**) Schematic workflow for in vivo addback experiment using R878H mouse bone marrow cells. (**B**) ProteinSimple Western blot showing DNA methyltransferase protein abundance in R878H lineage depleted bone marrow cells 2 days after transduction (Pre) and 1 month after transplantation of GFP^+^ bone marrow cells. (**C**) Heatmap showing the methylation values for individual samples of 4453 DMRs defined by comparing WGBS data from WT (*n* = 9) versus R878H (*n* = 6) samples. Values for the same DMRs were plotted passively for R878H samples that were retrovirally transduced, transplanted to recipient mice, and harvested at 1 month after transplantation. WGBS was performed on purified GFP^+^ cells from each mouse. (**D**) Mean methylation values for the R878H DMRs in WT, R878H, and transduced R878H samples from WGBS data used in (C). (**E**) Mean methylation values of CpGs from WGBS of WT, R878H, and addback samples. Mean values for all CpGs and DMR associated CpGs in annotated regions of the genome are shown.

To determine whether overexpression of DNMT3A1 or DNMT3L in vivo could correct the methylation phenotype of the R878H mutation, we performed the experiment shown in Fig. 7A. Bone marrow cells were transduced with these vectors exactly as described above. Two days after transduction, 1 million total cells from each transduction (20 to 50% GFP^+^) were transferred to sublethally irradiated (6 Gy) C57BL/6 mice via retroorbital injection. Bone marrow samples were harvested 1 month after transplantation, and GFP^+^ cells were harvested for analysis. DNMT3A and DNMT3L protein levels in GFP^+^ cells were evaluated by Western blotting on the ProteinSimple platform (Fig. 7B), revealing persistent expression of both proteins in GFP^+^ cells at this time point. Methylation levels for the R878H DMRs in the addback samples are passively plotted in Fig. 7C, revealing near-complete remethylation with either vector after 1 month (Fig. 7D and fig. S7, A and B) in four independent biological replicates. DNMT3A and DNMT3L addback minimally altered global methylation levels (Fig. 7 and fig. S7C), but analysis of DMRs in all annotated genomic regions revealed near-complete correction of methylation values after 1 month in vivo (Fig. 7E).

**Fig. 8.**
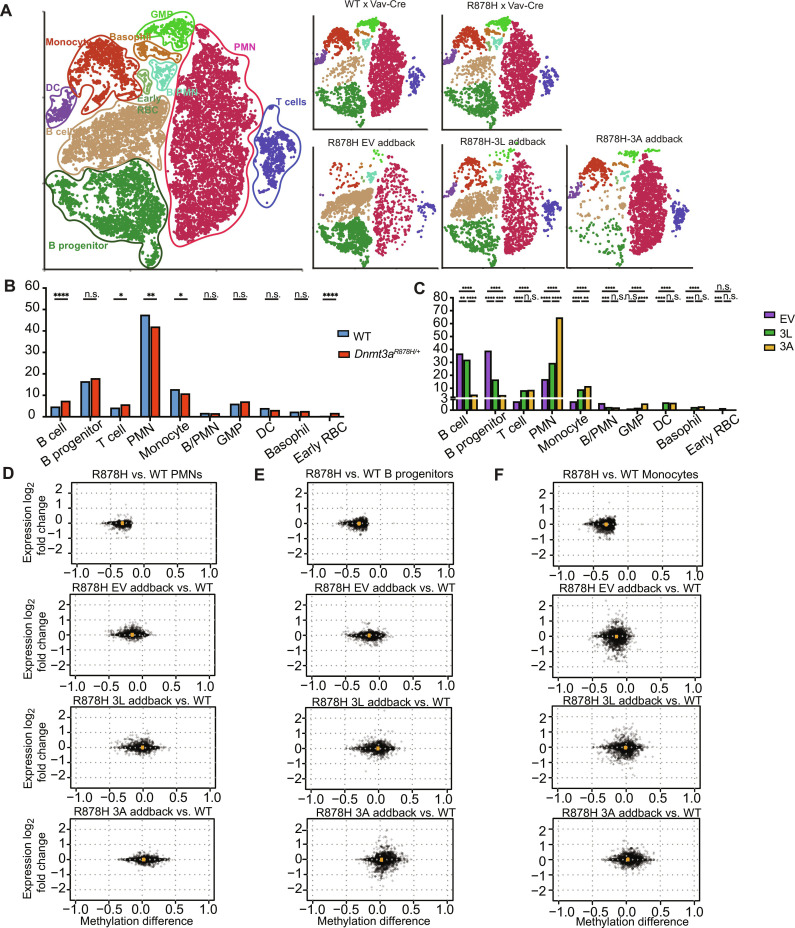
Effects of DNMT3L or DNMT3A addback on cellular populations and gene expression in R878H bone marrow. (**A**) t-SNE projections of merged and separate scRNA-seq data from unmanipulated WT versus R878H bone marrow cells from 2-month-old mice or from retrovirally transduced (EV, DNMT3L, or DNMT3A), GFP^+^ R878H bone marrow cells 1 month after transplantation (as in Fig. 7). Cell populations were assigned according to the Haemopedia algorithm and graphic-based manual review. B/Mac, biphenotypic B/macrophages; early red blood cells (RBCs) include MEPs and other erythroid progenitors. B progenitor cells include pro-B cells and pre-B cells. (**B** and **C**) Cell population distributions for WT, R878H, and GFP^+^ cells from each addback, using the scRNA-seq data in (A). Fisher’s exact test was used for statistical analysis. *P < 0.05; **P < 0.01; ***P < 0.001; ****P < 0.0001; n.s., not significant. (**D** to **F**) Top panel in each: Correlation between methylation and expression differences for individual genes within 1 kb of a DMR defined by the comparison of R878H and WT bone marrow. Panels below represent the differences between WT values at these DMRs, and the addback samples for EV, DNMT3L, or DNMT3A. *X* axes: Methylation differences at DMRs, showing the difference between WT and addback values for each DMR. *Y* axes: expression differences for genes within 1 kb of these DMRs, comparing the values in WT cells and addback cells. (D) Methylation versus expression differences in PMNs, (E) methylation versus expression differences in B cell progenitors, and (F) methylation versus expression differences in monocytes.

To further define the functional consequences of overexpressing DNMT3A and DNMT3L in hematopoietic cells, we performed scRNA-seq on the GFP^+^ in vivo *Dnmt3a^R878H/+^* addback samples 1 month after transplantation (Fig. 8A), as well as unmanipulated WT and *Dnmt3a^R878H/+^* whole–bone marrow cells from littermate-matched, 8-week-old mice. The in vivo addback samples (EV, DNMT3A, and DNMT3L) were all subjected to ex vivo cytokine–driven expansion and retroviral transduction, and then transplanted into sublethally irradiated mice; 1 month later, the transduced, GFP^+^ cells were purified for scRNA-seq. These cells are therefore not equivalent to the unmanipulated marrow controls from WT versus *Dnmt3a*^R878H/+^ mice. Regardless, several key observations can be made from these data. First, persistent overexpression of the human *DNMT3A* and *DNMT3L* mRNAs for 1 month was confirmed in the scRNA-seq data (fig. S8, A and B). Second, scRNA-seq data from unmanipulated bone marrow cells of 2 month old WT versus R878H mice revealed a small increase in bone marrow B cells with the R878H sample and a small decrease in mature monocytes and polymorphonuclear cells (PMNs; Fig. 8B) (*39*); these data are similar to that of the bone marrow of mice with a germline *Dnmt3a*^R878H/+^ mutation (*16*). Third, the control (EV) in vivo addback R878H sample exhibited even more prominent skewing toward B cell progenitors and B cells and away from mature PMNs and monocytes (Fig. 8, A and C). The more notable lineage shifts (compared to the unmanipulated R878H marrow) may be related to the proliferative stress caused by transplantation (*8*). Fourth, the addback of DNMT3L and DNMT3A both caused a partial reversal of this abnormal lineage shift, reducing B cells and increasing the proportion of mature PMNs and monocytes in the addback samples (Fig. 8, A and C). Last, we compared global patterns of gene expression for genes within 1 kb of *Dnmt3a*^R878H/+^ DMRs, using the addback scRNA-seq data for PMNs, B cell progenitors, and monocytes, because these populations were abundant in all samples (Fig. 8, D to F). The methylation levels at DMRs in the R878H samples were nearly completely corrected with DNMT3L or DNMT3A addback in vivo (Fig. 7 and fig. S7, for global CpG remethylation). However, the expression of genes within 1 kb of the R878H DMRs were small and most were nonsignificant; there were < 50 differentially expressed genes in each compartment for the comparison of WT versus *Dnmt3a*^R878H/+^ mice, and the global expression patterns of these genes were not notably altered with addback (Fig. 8, D to F). We extended this analysis to all genes within 10 kb of DMRs, with similar results (fig. S9).

Last, to determine whether long-term overexpression of DNMT3A1 or DNMT3L might have adverse consequences in WT hematopoietic cells, we performed in vivo addback experiments using an EV or *DNMT3A* or *DNMT3L* vectors in WT mouse bone marrow cells, exactly as described in Fig. 7. One and 2 months after transplantation, purified GFP^+^ cells were subjected to WGBS. DMRs were identified by comparing EV GFP^+^ cells (from both the 1- and 2-month samples) with the 1-month samples from the DNMT3A and DNMT3L addback. Only 39 DMRs were identified, of which 34 were hypermethylated (fig. S10A and table S7). Evaluation of the methylation status of these DMRs in the samples harvested at 2 months revealed similar findings at most sites. The nearest neighbor genes for these DMRs were not dysregulated in bulk RNA-seq datasets for WT versus R882H bone marrow samples, suggesting that the expression of these genes was not affected by this mutation. Last, we performed complete blood counts on mice transplanted with marrows transduced with EV, DNMT3A, or DNMT3L after 1 or 2 months and noted only small changes (fig. S10B).

## DISCUSSION

Here, we describe the DNA methylation phenotypes of unmanipulated primary bone marrow samples from mice that are deficient for one or both of the de novo DNA methyltransferases or expressing the *Dnmt3a^R878H/+^* mutation. In all cases, highly efficient floxing was induced with *Vav1-Cre*. Focal, canonical hypomethylation phenotypes in similar areas of the genome were observed in all the mouse models, with different grades of severity depending on genotypes (DKO > 3a KO > R878H > 3b KO). Using a retroviral overexpression system, we established an efficient method to restore the methylation values of DMRs in each deficiency state in 14 days. Overexpression of active full-length *DNMT3A1* remethylated DMRs in all DNA methyltransferase–deficient bone marrow cells. *DNMT3B1* overexpression fully restored *Dnmt3b* KO DMRs and partially restored the methylation phenotype in other models. *DNMT3B3* was inactive in 3a and DKO mice, but its addback was able to reverse the hypomethylation phenotype in 3b KO mice, suggesting that it acts to increase the activity of DNMT3A in this setting. Because DNMT3L also is known to augment the activity of DNMT3A and DNMT3B during early embryogenesis, we evaluated its ability to augment the activity of the R878H mice; short-term overexpression of *DNMT3L* nearly completely corrected the hypomethylation phenotype of these cells within weeks, without causing inappropriate hypermethylation. Last, addback of *DNMT3L* or *DNMT3A* in vivo also had functional consequences for hematopoietic differentiation, partially correcting a differentiation block in terminal myeloid maturation associated with the *Dnmt3a*^R878H^ mutation.

Previous studies have suggested that DNMT3B has distinct functions from DNMT3A (*23*, *40*) and that it methylates different regions of the genome during embryogenesis. However, in both human and mouse hematopoietic cells, the dominant splice isoform detected is DNMT3B3, which is missing a portion of the catalytic domain and therefore inactive as a DNA methyltransferase (*8*, *9*, *23*). The hematopoietic cells of 3b KO mice had far fewer DMRs than 3a KO mice, and the level of hypomethylation was less severe. However, the locations of these DMRs overlapped almost completely with the 3a KO DMRs, suggesting that these two enzymes may synergize to provide the de novo methyltransferase activity of early hematopoietic cells. Addback experiments strongly suggested that this synergy is a consequence of DNMT3B augmenting the activity of DNMT3A, probably by forming a functional heterodimer and/or heterotetramers with DNMT3A, as previously suggested (*25*, *26*, *41*). We therefore hypothesize that the 3b KO DMRs may actually reflect a small decrease in the activity of DNMT3A, a hypothesis that is also supported by the similarity of the methylation phenotypes of 3b KO and R878H mice (see Fig. 1).

The ability of DNMT3B3 to augment the function of DNMT3A is reminiscent of the chaperone function of DNMT3L in early embryogenesis (*25*). DNMT3L not only can substantially enhance the methyltransferase activity of DNMT3A in vitro (*38*) and in tissue culture cells (*14*) but also can increase the binding of DNMT3A to its DNA targets by changing the conformation of DNMT3A multimers. DNMT3L can also recognize modified histone marks (including unmethylated H3K4 and H3K36me3-enriched regions) (*25*, *42*). Because DNMT3L is epigenetically silenced in adult hematopoietic cells (including AML cells) (*9*), it probably does not contribute to the function of DNMT3A in this compartment. However, if it could be reactivated genetically or pharmacologically, then it may have the potential to remethylate DNA if cells contain a residual WT *DNMT3A* allele.

The overexpression of *DNMT3A1* or *DNMT3L* in *Dnmt3a*^R878H/+^ mouse bone marrow cells accurately restored the methylation landscape of these cells within weeks. Inappropriate hypermethylation was not detected at promoters or CpG islands nor at regions where DNMT3A is not normally active (low methylated regions). Hypermethylated regions were not detected genome-wide nor in any annotated regions of the genome (*16*). Furthermore, overexpression of *DNMT3A1* or *DNMT3L* in WT bone marrow cells in vivo for 1 or 2 months caused only 39 DMRs in the entire genome and did not significantly alter the blood counts of these mice (fig. S10). These data suggest that additional (as yet undefined) factors may restrict the ability of DNMT3A to methylate some specific CpGs; further, an autoregulatory loop was suggested by the downregulation of endogenous *Dnmt3a* and *Dnmt3b* expression with the in vivo addback of *DNMT3A* or *DNMT3L* for 1 month (see fig. S8A). Alternatively, it is possible that hypermethylation is not tolerated and that cells with this phenotype are selected against. However, we did not find any evidence for selection against DNMT3A overexpressing cells in any in vitro addback experiment (based on stable levels of GFP^+^ cells overexpressing *DNMT3A1* for 2 weeks after transduction). Because the correction of hypomethylated DMRs was complete within weeks for *DNMT3A1* or *DNMT3L* addback, and because the maintenance methyltransferase, DNMT1, would be expected to maintain a corrected methylation phenotype, it may be possible to repair hypomethylation phenotypes with short-term bursts of excess DNMT3A activity mediated by DNMT3A or DNMT3L (*16*, *43*). If persistent *DNMT3A* loss-of-function (and its associated hypomethylation phenotype) is essential for the survival of fully transformed AML cells, then transient reactivation of DNMT3L could potentially allow for the remethylation of these cells, perhaps causing differentiation and or growth arrest. Additional studies to address this possibility are in progress.

In summary, these observations suggest a potentially relevant strategy for reactivating DNMT3A function: reexpression of *DNMT3L*. The rapidity and accuracy of remethylation caused by short-term reexpression of *DNMT3L* do not appear to be deleterious in WT cells, nor in non-transformed hematopoietic cells expressing the *Dnmt3a*^R878H^ mutation. Additional experiments will need to be required to determine whether fully transformed AML cells with *DNMT3A*^R882^ mutations are “addicted” to their DNA hypomethylation phenotype, and whether its correction will slow the growth of these cells or change their developmental fate (*9*, *19*, *20*, *44*). However, therapies that are capable of reactivating the expression of *DNMT3L* in human cells [including HDAC inhibitors and/or HMAs (*10*–*13*)] have already shown some promise in treating AML patients, and the observations noted here should be explored in future studies.

## MATERIALS AND METHODS

### Mouse models

All mice were in the C57BL/6J background. *Dnmt3a^−/−^ and Dnmt3a^+/−^* mice were generated by crossing Cre/loxP conditional mutant mice *Dnmt3a^tm3.1Enl^* from the Mutant Mouse Regional Resource Centers repository (MMRRC strain name: B6;129S4- *Dnmt3a^tm3.1Enl^*/Mmnc) (*45*) and Vav1-iCre mice [the Jackson Laboratory, B6.Cg-Commd10Tg(Vav1-icre)A2Kio/J]. *Dnmt3b^−/−^* mice were generated by crossing Cre/loxP conditional mutant mice *Dnmt3b^tm5.1Enl^* from the Mutant Mouse Regional Resource Centers repository [MMRRC strain name: B6.129S4(Cg)-*Dnmt3b^tm5.1Enl^*/Mmnc] (*46*) with Vav-iCre mice. *Dnmt3a^−/−^ × Dnmt3b^−/−^* were generated with the *Dnmt3a^tm3.1Enl^*, *Dnmt3b^tm5.1Enl^*, and Vav1-iCre mice. Mice with *Dnmt3a^R878H/+^* mutation (mouse homolog of the human *DNMT3A^R882H^* mutation) in bone marrow cells were generated by crossing Cre/loxP conditional mutant mice C57BL/6-*Dnmt3a^tm1Rlvn^*/GrynvJ (the Jackson Laboratory, strain no. 031514) (*36*) with Vav1-iCre mice. These mice were provided by O. Guryanova and R. Levine. Whenever possible, littermate controls were used for all experiments. All mouse studies were done in accordance with institutional guidelines and were approved by the Animal Studies Committee at Washington University.

### Retroviral transductions

Retroviruses were generated by transfecting GP2-293 T cells (Takara Bio) with murine stem cell virus (MSCV)–internal ribosomal entry site–GFP retroviral plasmids having either no insertion, full-length WT *DNMT3A1*, *DNMT3A^R882H^* [generated by QuickChange II site directed mutagenesis (Agilent)], full-length DNMT3B1, DNMT3B3, or DNMT3L cDNAs using TransIT-LT1 (Muris Bio). The DNA sequences of all plasmids used in these studies were verified by whole plasmid sequencing performed by Plasmidsaurus. We harvested supernatants at 24 and 48 hours after transfection by filtering through 0.45-μm filter (Corning) and added the filtered viral supernatants to six-well non-tissue–treated plates coated with Retronectin (Takara Bio; 5 μg/ml, 24 hours at 4°C). The plates containing viral supernatants were spun at 2500*g* for 90 min at 32°C. One to 2 million lineage depleted cells in bone marrow transplant media were added to each well of the six-well plate after the viral supernatants were discarded and were spun at 1000 rpm for 7 min at 32°C. Cells were scraped from the plates and transduced again with the 48-hour harvested viral supernatants.

### Bone marrow transplantation

Femur, tibia, pelvic, and humerus-derived bone marrow cells were harvested from mice in RPMI 1640 media (Gibco) with 15% FBS (Atlanta Biologicals). Bone marrow cells were treated with 1× ammonium chloride/KCl red blood cell lysis buffer and resuspended in transplant media [RPMI 1640 with 15% FBS, 1% penicillin-streptomycin (Gibco), and cytokines (mouse FLT3L, 50 ng/ml; mouse Kit Ligand, 100 ng/ml; mouse IL-3, 6 ng/ml; and mouse TPO, 10 ng/ml)] for ex vivo cultures.

Ly45.1 C57BL/6 recipients were purchased from Charles River Laboratories. Sublethal irradiation was performed by exposing mice to 6 Gy as a single dose. Transduced bone marrow cells were resuspended in phosphate-buffered saline (PBS) buffer at 1 million cells/100 μl. Transplantation was performed by injecting 1 million transduced bone marrow cells into the retro-orbital venous sinus. Mice were on antibiotic-supplemented water (sulfamethoxazole and trimethoprim) for 2 weeks after transplantation. Peripheral blood was obtained by piercing retro-orbital veins with heparinized capillary tubes (Thermo Fisher Scientific) after anesthesia with isoflurane.

### WGBS and analysis

DNA was isolated with the QIAamp DNA Micro Kit (QIAGEN, 56304). Input DNA (50 to 100 ng) was bisulfite converted with the DNA Methylation Gold Kit (Zymo Research). Whole-genome bisulfite–converted sequencing libraries were generated with an Accel NGS Methyl-Seq DNA library kit (Swift Biosciences, no. 30096). Indexed sequencing was performed on Illumina NovoSeq 6000 instruments. Reads were mapped using biscuit, and DMRs were called using metilene (*47*) as previously described (*9*, *15*, *16*, *19*). The workflow for WGBS data is described in detail at https://github.com/genome/analysis-workflows/blob/v1.5.0_fix_1/definitions/pipelines/bisulfite.cwl. All data for WGBS studies were deposited in the Short Read Archive (SRA) under BioProject PRJNA1008414.

### ProteinSimple Western blotting

Western blotting was performed on the Jess ProteinSimple platform. Lysates were generated by sonicating cells (100,000 cells/μl) in 1× NuPAGE lithium dodecyl sulfate (LDS) sample buffer (Invitrogen) with the Sonifier 450 (Branson) setting Output Control to 3, 20% pulse for 20 s. Lysates were clarified at 13,000 rpm for 10 min at 4°C. Samples were blotted with rabbit anti-DNMT3A (Cell Signaling Technology, D23G1; 1:100 dilution), rabbit anti-DNMT3B (Cell Signaling Technology, D7O7O; 1:100 dilution), mouse anti–Beta-Actin (Novus, NB600-532S; 1:100 dilution), rabbit anti-DNMT3B (Abcam, ab2851; 1:50 dilution), rabbit anti-DNMT3L (Abcam, ab194094; 1:50 dilution), horseradish peroxidase (HRP)–conjugated anti-Rabbit (ProteinSimple), near-infrared (NIR)-conjugated anti-mouse (ProteinSimple), and HRP-conjugated anti-mouse (ProteinSimple). Protein abundance on blots was quantified by Compass for Simple Western software.

### scRNA-seq and analysis

scRNA-seq was performed as previously described (*16*). Libraries were generated with the 10X Genomics system Chromium Single Cell 5′ library Kit (v2) and sequenced on Illumina NovaSeq machines. scRNA-seq data were aligned using the Cell Ranger pipeline. Cells were annotated on the basis of the Haemopedia expression atlas (*37*). Cell populations were further defined by manual review on Partek Flow software, based on gene expression profiles. Differentially expressed genes were defined by the analysis of variance (ANOVA) algorithm with Partek Flow software. All RNA-seq data were deposited in the SRA (under BioProject PRJNA1008414).

### Total RNA-seq and identification of transcript isoforms

Total RNA-seq of *Dnmt3a*^WT^ and *Dnmt3a^R878H/+^* mice was performed using the Illumina TruSeq Stranded Total RNA Library Kit on deoxyribonuclease-treated RNA and sequenced on the NovaSeq 6000 platform, with 2 × 151-bp reads.

*Dnmt3a* and *Dnmt3b* isoforms (*24*, *48*) were defined using the following approach. The *Dnmt3a1* (Ensembl transcript ENSMUST00000020991) and *Dnmt3a2* (Ensembl transcript ENSMUST00000172689) isoforms were differentiated using bulk RNA-seq and based on RegTools analysis [PubMed IDentifier (PMID): 36949070] of splice junction usage at exon 7 of the longer *Dnmt3a1* transcript. Reads splicing from the 3′ untranslated region of the internal promotor to exon 7 supported *Dnmt3a2*, while reads splicing from exon 6 to exon 7 supported the full-length *Dnmt3a1*. Isoform usage percentage was calculated as the number of reads supporting one junction over the total number of reads in both junctions. *Dnmt3b* isoform usage was calculated similarly (*24*), using the count of splice junctions that skip exons 22 and 23 to identify the inactive *Dnmt3b3* isoform (ENSMUST00000088976/ENSMUST00000103150) and the mean count of splice junctions that connect exons between 21 and 24 to determine the *Dnmt3b1* counts (ENSMUST00000109774/ENSMUST00000081628). A Fisher’s exact test on pooled counts was used to test the difference in proportions between WT and R878H mice.

### Recombinant DNMT3A1 production

This proceedure was performed as previously described (*8*). Six million HEK293T cells were plated on 15-cm plates in RPMI 1640 (Gibco) [including 10% FBS (Atlanta Biologicals) and 1% penicillin-streptomycin (Gibco)] overnight. Total 6xHis-DNMT3A-FLAG expression plasmids (25 μg), 6xHis-DNMT3A-FLAG (12.5 μg) with 12.5 μg of DNMT3B3-myc expression plasmids, or 6xHis-DNMT3A-FLAG (12.5 μg) with 12.5 μg of DNMT3L-myc expression plasmids were transfected with calcium-phosphate transfection methods on the following day. Medium was changed without disturbing the cells 16 hours after transfection. Cells were harvested in cold PBS at 48 hours after transfection and resuspended at 10 million cells/ml in lysis buffer [20 mM sodium phosphate (pH 7.65), 150 mM NaCl, and 20 mM imidazole]. Cell lysates were prepared by sonication, using a Sonifier 450 (Branson) (8 s, Output Control to 3.5, constant, repeated three times). Lysates were clarified at 13,000 rpm for 10 min at 4°C. The supernatants were passed through 0.45-μm filters (Corning) and loaded into a 1-ml HisTrap HP column (Cytiva) and washed with 10 ml of buffer [20 mM sodium phosphate (pH 7.65), 150 mM NaCl, and 50 mM imidazole]. Proteins were eluted in elution buffer [20 mM sodium phosphate (pH 7.65), 150 mM NaCl, and 400 mM imidazole] and dialyzed into 20 mM Hepes (pH 7.65), 30 mM NaCl, and 1 mM EDTA with 10% glycerol. DNMT3A protein abundance was measured using Western blotting using the ProteinSimple Jess system. Purified proteins were stored at −80°C in individual aliquots and never subjected to more than one freeze-thaw cycle.

### Mass spectrometry and data analysis

Purified samples with recombinant DNMT3A were digested for 2 hours at 30°C in a ThermoMixer with gyration at 750 rpm. Trypsin (1 μg) was added, and the samples were incubated overnight at 30°C in the ThermoMixer, gyrating at 750 rpm. Tryptic peptides were then transferred to a fresh tube, the bead samples were washed with an additional 50 μl of ammonium bicarbonate (ABC) buffer, and the wash was combined with the peptides. Residual detergent was removed by ethyl acetate extraction. In preparation for desalting, peptides were acidified to pH 2 with 1% trifluoroacetic acid (TFA) final concentration. The peptides were desalted using two micro-tips (porous graphite carbon, BIOMETNT3CAR) (Glygen) on a Beckman robot (Biomek NX). The peptides were eluted with 60% MeCN in 0.1% TFA and dried in a Speed-Vac (Thermo Scientific, Model No. Savant DNA 120 concentrator) after adding TFA to 5%. The peptides were dissolved in 20 μl of 1% MeCN in water. An aliquot (10%) was removed for quantification using the Pierce Quantitative Fluorometric Peptide Assay kit (Thermo Fisher Scientific, cat. no. 23290). The remaining peptides were transferred to autosampler vials (Sun-Sri, cat. no. 200046), dried, and stored at −80°C for liquid chromatography–mass spectrometry (MS) analysis on timsTOF Pro mass spectrometer. Peptide counts were normalized according to the size of each protein, and relative abundance was calculated after normalization to detected levels of DNMT3A as follows: Data from the mass spectrometer were converted to peak lists using Proteome Discoverer (version 2.1.0.81, Thermo Fisher Scientific). The MS2 spectra with charges +2, +3, and +4 were analyzed using Mascot software (Matrix Science, London, UK; version 2.8.1). The searches were performed with a mass tolerance of 20 parts per million for both precursor and fragment ions. Tandem MS spectra were set up to search against a UniProt database of human proteins (January 2023; 20,423 entries) and common contaminant proteins (version 1.0; January 2012; 116 entries), assuming that the digestion enzyme was trypsin/P with a maximum of four missed cleavages allowed. Carbamidomethylation of cysteine was specified as a fixed modification. Deamidation of asparagine and glutamine, formation of pyro-glutamic acid from N-terminal glutamine, acetylation of protein N terminus, and oxidation of methionine were specified as variable modifications. Peptide spectrum matches (PSMs) were filtered at 1% FDR by searching against a reversed database and the ascribed peptide, and protein identities were accepted.

The precursor intensities were converted to logarithmic ratios at base two (log2FC), relative to the average precursor intensity across all samples. Under each sample, Dixon’s outlier removals were carried out recursively for peptides with greater than two identifying PSMs. The median of the PSM log2FC that could be assigned to the same peptide was taken to represent the ratios of the incumbent peptide. The median of the peptide log2FC was taken to represent the log2FC of the inferred protein. To align protein ratios across samples, likelihood functions were first estimated for the protein log2FC using finite mixture modeling (R package: mixtools::normalmixEM), assuming two-component Gaussian mixtures. The distributions of log2FC were then aligned so that maximum likelihood was centered at zero for each sample. Scaling normalization was performed to standardize the protein log2FC across all samples. To reduce the influence of outliers, the values between the 5th and 95th percentile of log2FC and 5th and 95th percentile of intensity were used in the calculations of SDs. Following the normalization of protein log2FC, protein intensities were calibrated by an anti-logarithmic conversion of 2*^d^*, where *d* is the distance of log2FC before and after the ratio normalization. The obtained protein intensities were used for protein stoichiometry predictions. These data are available at ProteomeXchange (accession number: PXD044808).

### In vitro methyltransferase assay

Equivalent amount of immunoreactive DNMT3A, defined by quantification with Western blotting (DNMT3A, DNMT3A/DNMT3B3, and DNMT3A/DNMT3L), was incubated with 5 μM ^3^H-labeled S-Adenosyl methionine (SAM) (PerkinElmer) and 1 μg of pcDNA3.1 (Invitrogen) in 20 mM Hepes (pH 7.65), 30 mM NaCl, 1 mM EDTA, 0.5 mM dithiothreitol, and bovine serum albumin (0.2 mg/ml) at 37°C for 20 hours. Samples were spotted on filters in NucleoSpin Gel and PCR clean-up kits (Macherey-Nagel) and washed twice with the washing buffer provided in the kit. The columns were dried by centrifugation at 13,000 rpm for 2 min, and incorporated radioactivity was measured by liquid scintillation (Beckman Coulter LS 6500).

### Co-immunoprecipitation assays

Three million K562 cells were electroporated with 4 μg of a plasmid expressing a human *DNMT3L*-myc–tagged cDNA alone or with 4 μg of plasmids expressing either a human *DNMT3A* cDNA or a mouse *Dnmt3a* cDNA, using the Lonza SF cell line kit (Lonza, V4XC-2024). After electroporation, cells were cultured in RPMI 1640 media with 10% FBS and harvested 24 later in 500 μl of Pierce IP lysis buffer (Thermo Fisher Scientific, product no. 87787), followed by sonication with Sonifier 450 (Branson) setting Output Control to 3, 30% pulse, for 20 s. Lysates were clarified at 13,000 rpm for 10 min at 4°C. Myc-binding beads were prepared by adding 10 μl of a conjugated anti–myc-tag antibody (Cell Signaling Technology, no. 9B11) with 50 μl of prewashed Pierce Protein A/G Magnetic Beads (Thermo Fisher Scientific, product no. 88802) in 250 μl of Pierce IP lysis buffer for 1 hour at 4°C. Supernatants were removed, and then 500 μl of the clarified cell lysates were added to each tube, rotating the mixture gently overnight at 4°C. Beads were purified using a magnetic stand and washed three times with 500 μl of 1× tris-buffered saline (Thermo Fisher Scientific, product no. 28360). Last, 100 μl of LDS sample buffer was added to the beads, and proteins were eluted at 99°C for 10 min; the supernatants were then analyzed by Western blotting on the ProteinSimple platform. Antibodies used for Western blotting included anti–myc-tag antibody (Cell Signaling Technology, no. 9B11) for Myc-tagged DNMT3L and anti-DNMT3A antibody (Cell Signaling Technology, no. D23G1) for DNMT3A, which cross-reacts with mouse and human DNMT3A.

### Statistical comparisons

All statistical comparisons were made using GraphPad Prism 5 software,

 except for statistics on sequencing data, which were calculated using the R statistical programming software as described above. Statistical tests used and significance cutoffs are detailed in each figure legend. All data represent means ± SD or SEM, as specified in the figure legends.
