# Supplementary material for: Rapid and accurate remethylation of DNA in Dnmt3a-deficient hematopoietic cells with restoration of DNMT3A activity
Source: Sci Adv. 2024 Jan 31;10(5):eadk8598. doi: 10.1126/sciadv.adk8598 (PMC10830114; doi:10.1126/sciadv.adk8598)
Supplement: Supplementary file 1 — Figs. S1 to S10 Legends for tables S1 to S7 [file sciadv.adk8598_sm.pdf]

Science Advances
AAAS

Supplementary Materials for

Rapid and accurate remethylation of DNA in *Dnmt3a*-deficient hematopoietic cells with restoration of DNMT3A activity

Yang Li *et al.*

Corresponding author: Timothy J. Ley, timley@wustl.edu

Sci. Adv. **10**, eadk8598 (2024)
DOI: 10.1126/sciadv.adk8598

The PDF file includes:

Figs. S1 to S10
Legends for tables S1 to S7

Other Supplementary Material for this manuscript includes the following:

Tables S1 to S7

A

Sample	Floxing efficiency
Dnmt3a_KO_1	96.49%
Dnmt3a_KO_2	100.00%
Dnmt3a_KO_3	74.86%
Dnmt3a_KO_4	95.56%
Dnmt3a-Dnmt3b_KO_1	93.96%
Dnmt3a-Dnmt3b_KO_2	100.00%
Dnmt3a-Dnmt3b_KO_3	87.76%
Dnmt3b_KO_1	99.14%
Dnmt3b_KO_2	99.41%
Dnmt3b_KO_3	96.62%
DNMT3A_R878H_1	90.91%
DNMT3A_R878H_2	100.00%
DNMT3A_R878H_3	100.00%

B

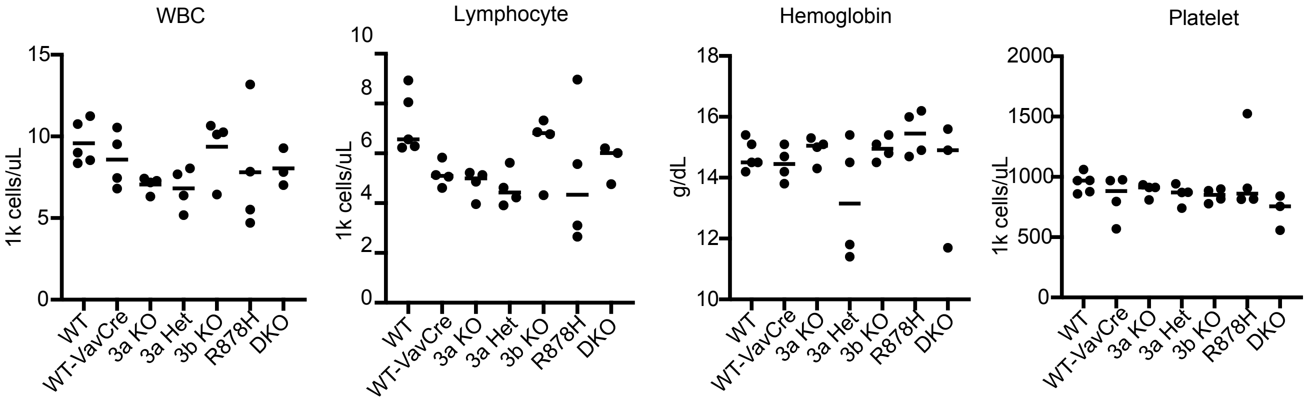

fig. S1. Floxing efficiency in *Dnmt* x Vav1-Cre bone marrow cells, and complete blood counts from mice. (**A**) Floxing efficiency for Dnmt deficient mice and R878H mice, deduced from WGBS data, or RNA-seq data from the bone marrow cells of each genotype. (**B**) Peripheral blood cell counts from 6–8-week-old, 3a KO, 3b KO, or DKO mice, or R878H mice. ANOVA testing was used to compare the blood counts among mice from different genotypes. None of the values were significant different among genotypes.

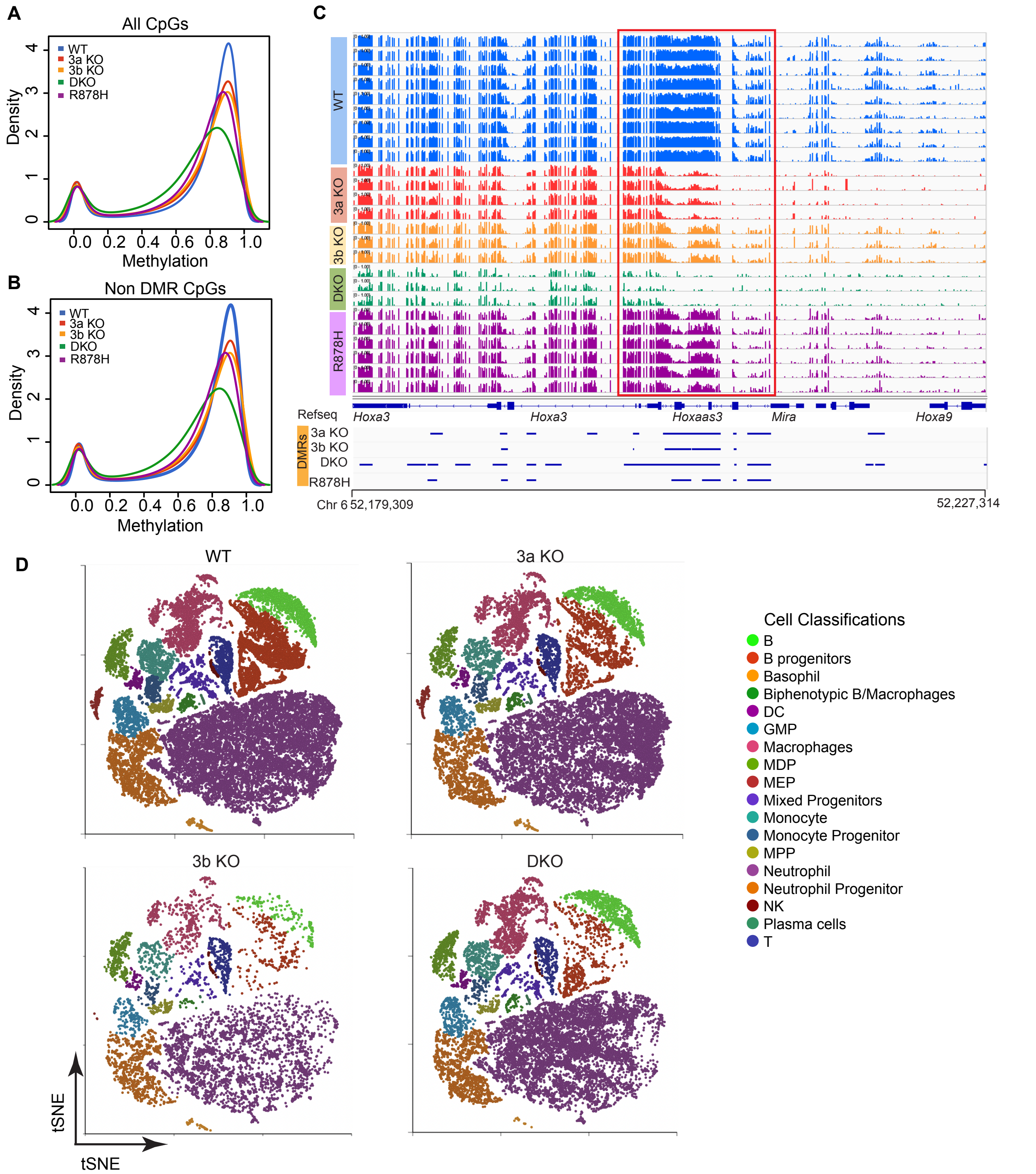
A
All CpGs
WT
3a KO
3b KO
DKO
R878H
Density
Methylation
B
Non DMR CpGs
C
WT
3a KO
3b KO
DKO
R878H
Refseq
Hoxa3
Hoxa3
Hoxaas3
Mira
Hoxa9
DMRs
Chr 6 52,179,309
52,227,314
D
WT
3a KO
3b KO
DKO
tSNE
tSNE
Cell Classifications
B
B progenitors
Basophil
Biphenotypic B/Macrophages
DC
GMP
Macrophages
MDP
MEP
Mixed Progenitors
Monocyte
Monocyte Progenitor
MPP
Neutrophil
Neutrophil Progenitor
NK
Plasma cells
T

fig. S2. Characterization of phenotypes in DNA methyltransferase deficient mouse bone marrow cells. (**A**) Density plots of mean methylation values for all CpGs for 3a KO, 3b KO, DKO, and R878H mice. (**B**) Density plot of mean methylation values from the non-DMRs CpGs from the same samples as in (A). (**C**) IGV view of a representative region of the *Hoxa* gene cluster. Each row represents a sample from a unique mouse. Methylation values for each CpG are shown as a bar ranging from 0-100% methylated in individual samples. Blue bars at the bottom indicate DMRs identified in this region. The red rectangle highlights the methylation phenotypes in different genotypes. (**D**) t-SNE projections of merged scRNA-seq data from whole bone marrow cells derived from each genotype (WT, 3a KO, 3b KO, and DKO, 6-8 weeks of age) (n=2 each). Cell populations were assigned according to the Haemopedia algorithm, and manual review. B progenitors include Pro-B cells and Pre-B cells; DC, dendritic cell; GMP, granulocyte-monocyte progenitors; MDP, monocyte dendritic cell progenitor; MEP, megakaryocyte erythrocyte progenitor; MPP, multipotent progenitor; NK, natural killer cells.

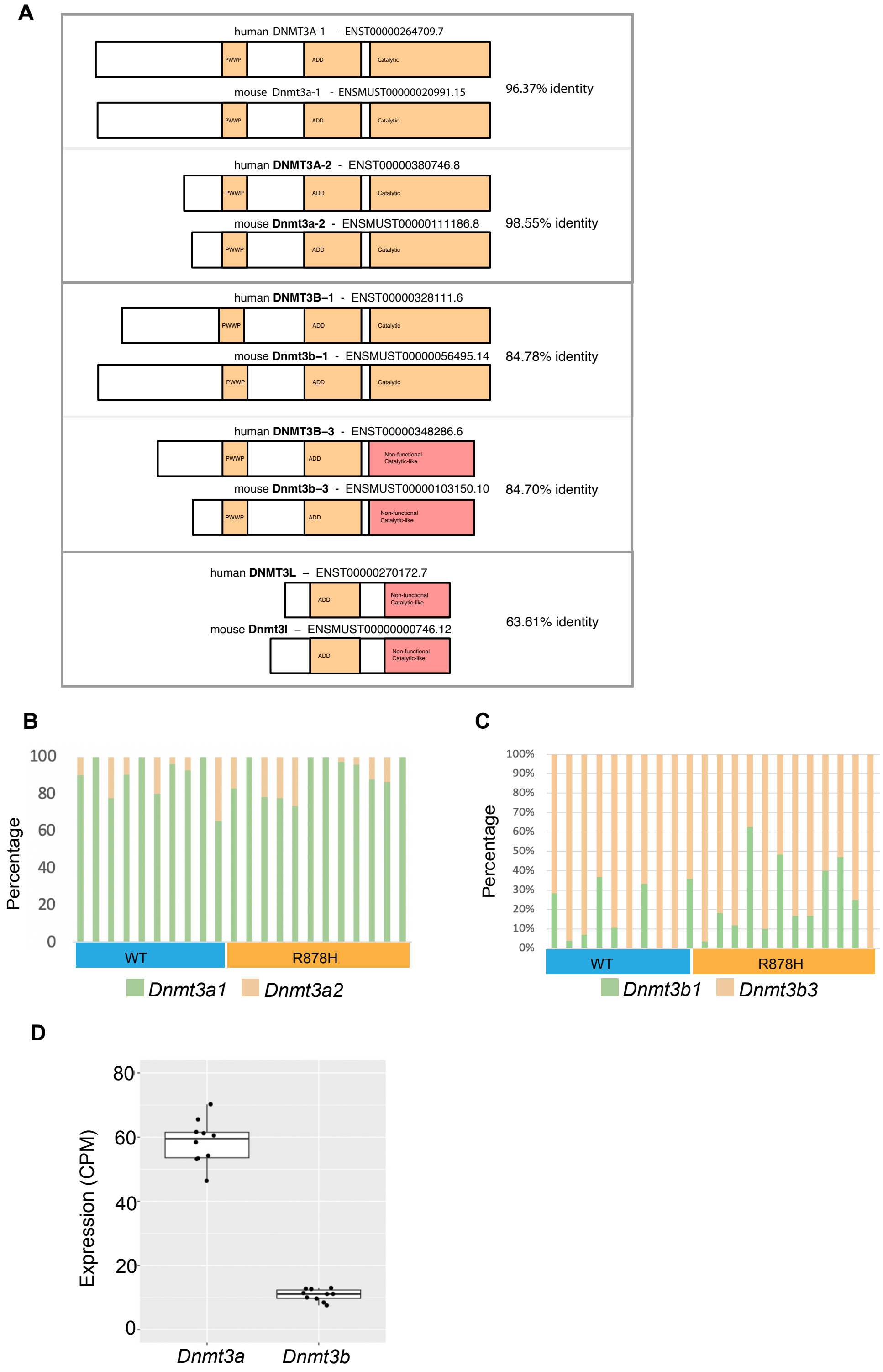
A
human DNMT3A-1 - ENST00000264709.7
PWWP
ADD
Catalytic
mouse Dnmt3a-1 - ENSMUST00000020991.15
96.37% identity
human DNMT3A-2 - ENST00000380746.8
mouse Dnmt3a-2 - ENSMUST00000111186.8
98.55% identity
human DNMT3B–1 - ENST00000328111.6
mouse Dnmt3b–1 - ENSMUST00000056495.14
84.78% identity
human DNMT3B–3 - ENST00000348286.6
Non-functional Catalytic-like
mouse Dnmt3b–3 - ENSMUST00000103150.10
84.70% identity
human DNMT3L – ENST00000270172.7
mouse Dnmt3l – ENSMUST00000000746.12
63.61% identity
B
Percentage
100
80
60
40
20
0
WT
R878H
Dnmt3a1
Dnmt3a2
C
Percentage
100%
90%
80%
70%
60%
50%
40%
30%
20%
10%
0%
WT
R878H
Dnmt3b1
Dnmt3b3
D
Expression (CPM)
80
60
40
20
0
Dnmt3a
Dnmt3b

fig. S3. Isoforms of *Dnmt3a* and *Dnmt3b* mRNA in mouse bone marrow samples from unmanipulated WT vs R878H samples. **(A)** Comparisons of the structure and homology of the major isoforms of DNMT3A, DNMT3B, and DNMT3L in humans and mice. (**B**) Bulk RNA-seq data was obtained from multiple unique mice with each genotype. Each bar represents data from one mouse. *Dnmt3a1* is the dominant isoform in both WT (n=10) vs. R878H (n=12) bone marrow samples. *Dnmt3a1* represents 88.6% and 88.5% *Dnmt3a* transcripts in WT and R878H respectively. (**C**) *Dnmt3b3* is the dominant isoform in both WT and R878H bone marrow samples*.* The differences between isoform use between the WT and R878H marrow samples were not significant for either gene. *Dnmt3b3* represents 78.2% and 78.1% *Dnmt3b* transcript in WT and R878H respectively. **(D)** Expression of total *Dnmt3a* vs. *Dnmt3b* mRNAs in WT mouse bone marrow samples (n=10), using the same data used in Panels B and C. *Dnmt3a* has a normalized expression value of 58.5 +/- 6.9 CPM (counts per million), vs. 10.8 +/- 1.8 CPM for *Dnmt3b*.

A

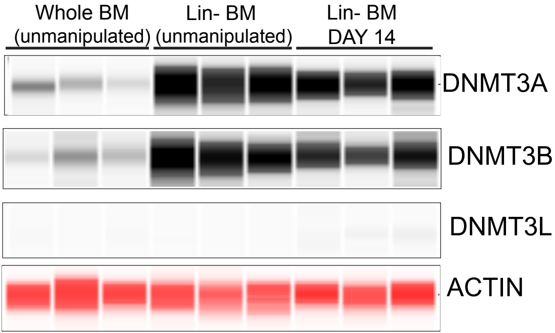

Whole BM
(unmanipulated)
Lin- BM
(unmanipulated)
Lin- BM
DAY 14
DNMT3A
DNMT3B
DNMT3L
ACTIN

fig. S4. DNMT3A, DNMT3B3, and DNMT3L abundance in unmanipulated vs. cultured WT bone marrow cells. Protein Simple "western blot" showing DNA methyltransferase protein abundance in WT whole bone marrow cells that were unmanipulated (i.e., obtained directly from a mouse, first 3 lanes), or lineage depleted cells from unmanipulated marrow (next 3 lanes), or lineage depleted cells that were cultured for 14 days in "transplant media" *ex vivo* (last 3 lanes). The levels of DNMT3A and DNMT3B3 cannot be directly compared, since the antibodies used to detect these proteins may have different affinities for their cognate targets. Lin- : lineage depleted.

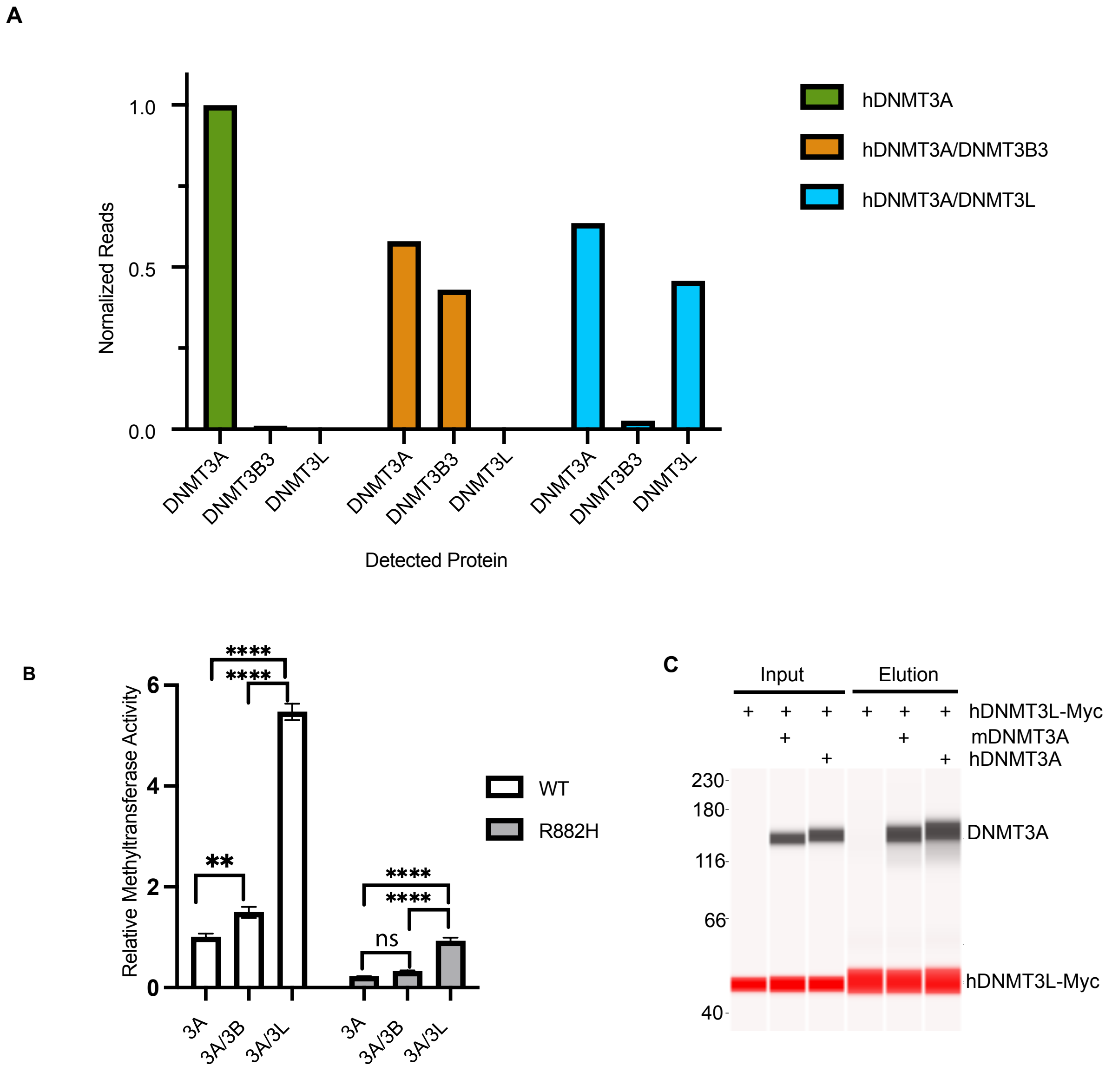
A
1.0
0.5
0.0
Normalized Reads
DNMT3A
DNMT3B3
DNMT3L
DNMT3A
DNMT3B3
DNMT3L
DNMT3A
DNMT3B3
DNMT3L
Detected Protein
hDNMT3A
hDNMT3A/DNMT3B3
hDNMT3A/DNMT3L
B
Relative Methyltransferase Activity
6
4
2
0
****
****
**
****
****
ns
3A
3A/3B
3A/3L
3A
3A/3B
3A/3L
WT
R882H
C
Input
Elution
+ + + + + + hDNMT3L-Myc
+ + mDNMT3A
+ + hDNMT3A
230
180
116
66
40
DNMT3A
hDNMT3L-Myc

fig. S5. DNMT3A activity is augmented by copurified DNMT3B3 or DNMT3L. (**A**) Normalized, relative abundance of DNMT3A, DNMT3B, and DNMT3L in purified protein preparations from transfected NIH3T3 cells. DNMT3A was purified on Nickel resin columns by virtue of a 5xHis tag fused in frame to human *DNMT3A1* cDNA. Plasmids containing human *DNMT3B3* and *DNMT3L* cDNAs did not have His tags. Their copurification was therefore caused by the "pulldown" of these proteins by DNMT3A. Protein abundance was quantified by mass spectrometry after tryptic digestion of the purified protein preparations. (**B**) *In vitro* methylation assay of purified DNMT3A1 (3A) from cells transfected with DNMT3A only, or cotransfected with DNMT3B (3A/3B), or DNMT3L (3A/3L). Cells transfected with *DNMT3B3* or *DNMT3L* only had no methyltransferase activity above background. Equal amounts of purified DNMT3A protein (defined by western blot-defined immunoreactive DNMT3A levels) were used to determine the methyltransferase activity. The activity of WT DNMT3A co-purified with DNMT3B3 is 1.49X compared with WT alone, and 5.47X when co-purified with DNMT3L. The activity of R882H DNMT3A co-purified with DNMT3B3 is 1.45X compared with R882H alone, and 4.16X when co-purified with DNMT3L. Hypothesis testing was performed using an ordinary one-way ANOVA test to compare each group of purified proteins' activity. ns p . 0.05; ** $p \leq 0.005$; **** $p \leq 0.001$. (**C)** Immunoprecipitation studies of Myc-tagged human DNMT3L in K562 human cells. Cells were co-electroporated with full length human or mouse *DNMT3A1* cDNAs, and MYC-tagged human *DNMT3L* cDNA as indicated, and 24 hours later, protein extracts were immunoprecipitated using an antibody directed against the MYC tag. Western blots were then performed with the Protein Simple system using an antibody specific for the MYC-tag to identify DNMT3L (red), or an antibody that interacts with either human or mouse

DNMT3A (black). Protein input levels are shown in the left panel ("Input"), and proteins pulled down with the anti-MYC antibody are shown in the right panel ("Elution").

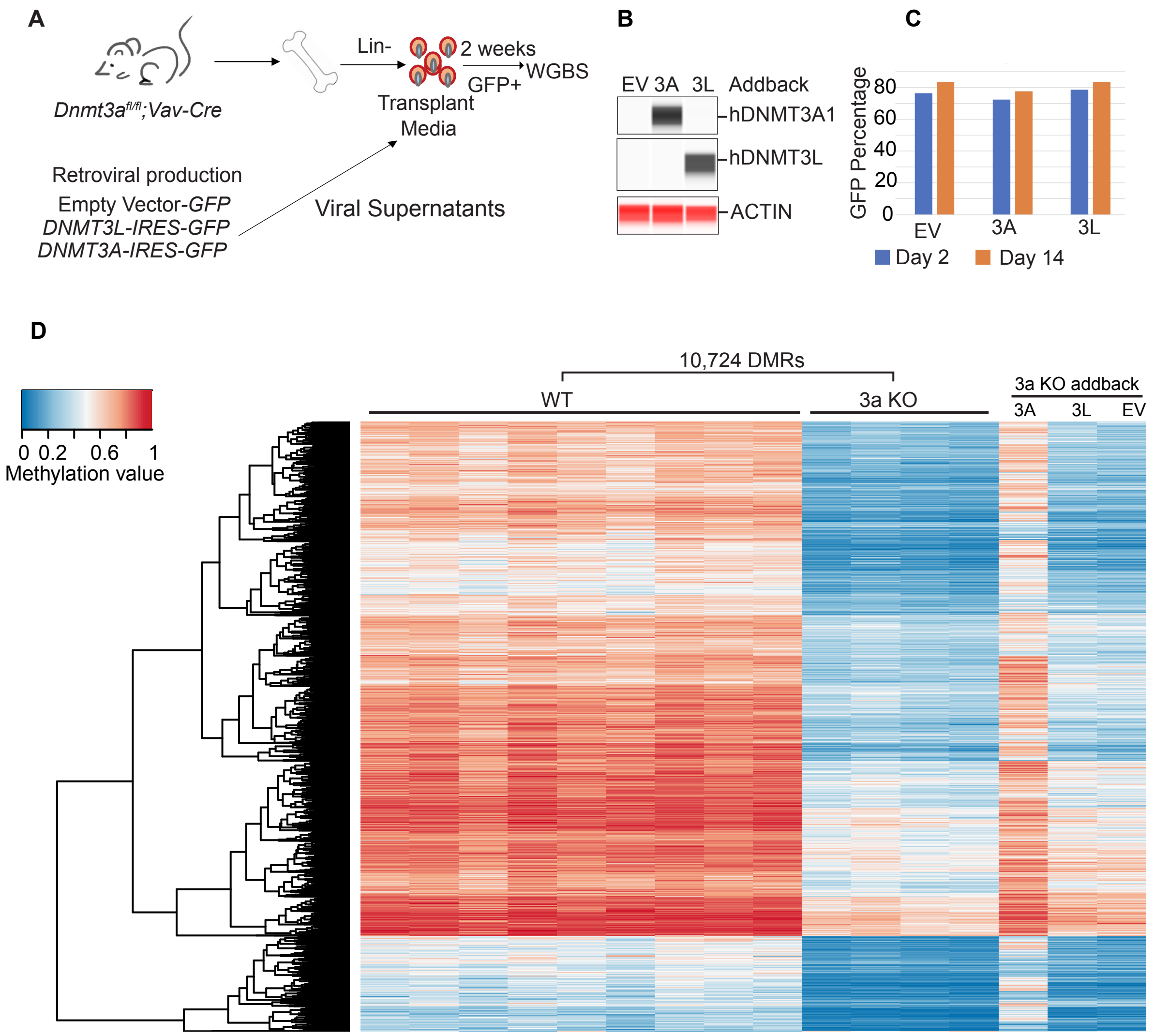

A
Dnmt3a$^{fl/fl}$;Vav-Cre
Lin-
2 weeks
GFP+
WGBS
Transplant
Media
Retroviral production
Empty Vector-GFP
DNMT3L-IRES-GFP
DNMT3A-IRES-GFP
Viral Supernatants
B
EV 3A 3L Addback
hDNMT3A1
hDNMT3L
ACTIN
C
GFP Percentage
0
20
40
60
80
EV
3A
3L
Day 2
Day 14
D
0 0.2 0.6 1
Methylation value
10,724 DMRs
WT
3a KO
3a KO addback
3A
3L
EV

fig. S6. Effects of retroviral addback *ex vivo* in primary *Dnmt3a*$^{flox/flox}$ x Vav1-Cre bone marrow cells. (**A**) Schematic workflow for an *ex vivo* addback experiment using *Dnmt3a*$^{-/-}$ primary mouse bone marrow cells. (**B**) Protein Simple western blot showing DNA methyltransferase protein abundance in lineage depleted 3a KO bone marrow cells two days post transduction. (**C**) Percentage of GFP+ 3a KO bone marrow cells at 2 days and 14 days post transduction. (**D**) Heatmap showing the methylation values for 10,724 DMRs (from Fig. 1A) defined by comparing WGBS data from WT (n=9) vs. 3a KO (n=4) samples. DNA methylation values for the same DMRs were plotted passively for purified GFP+ cells (i.e., transduced) from the indicated retroviral vectors into 3a KO bone marrow progenitors, after 14 days in liquid culture. “3A” = *DNMT3A1* cDNA vector. "3L" = *DNMT3L* cDNA vector. "EV" = empty vector. Note that DNMT3L has no intrinsic methyltransferase activity in bone marrow cells, in the absence of DNMT3A.

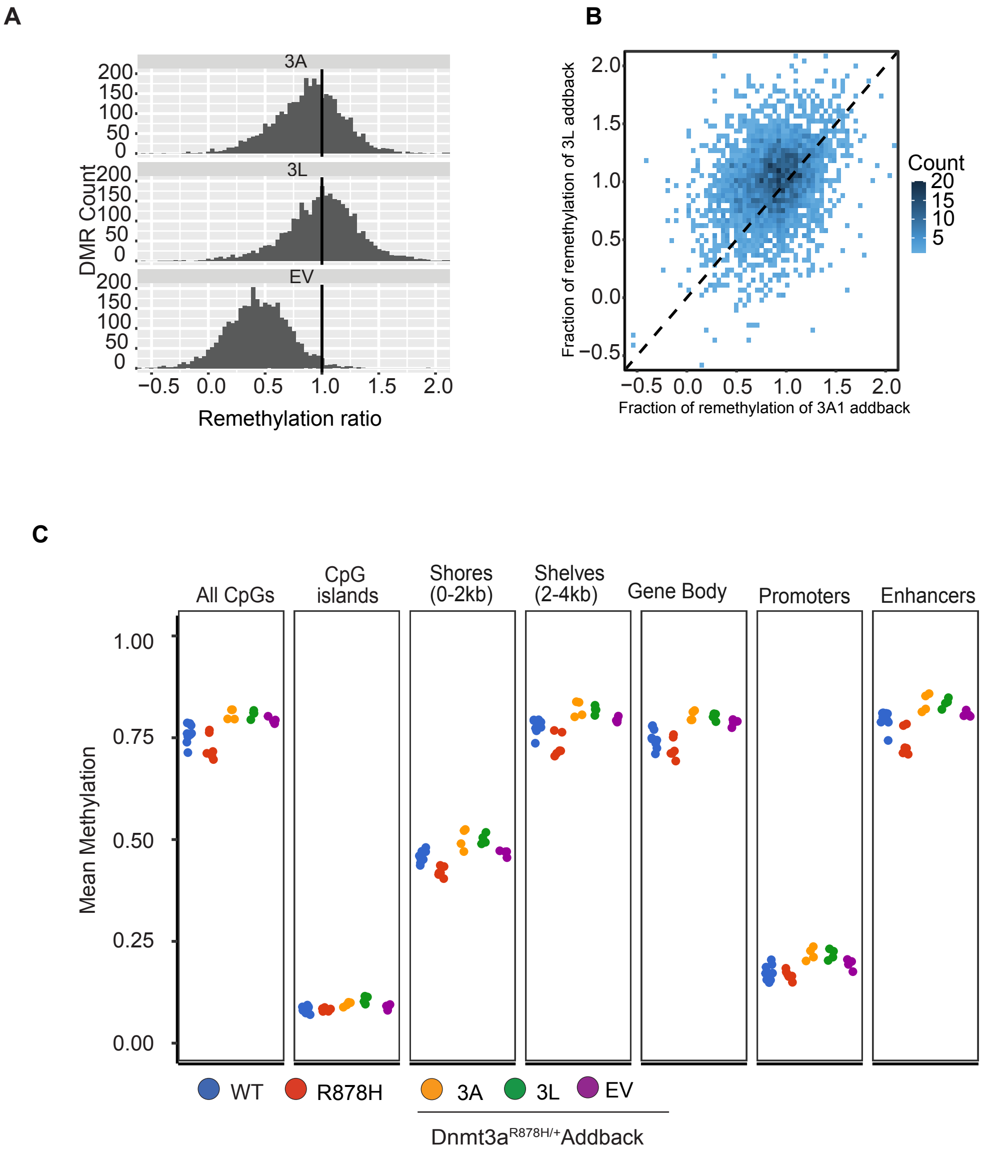
A
3A
3L
EV
DMR Count
Remethylation ratio
B
Fraction of remethylation of 3L addback
Fraction of remethylation of 3A1 addback
Count
20
15
10
5
C
All CpGs
CpG islands
Shores (0-2kb)
Shelves (2-4kb)
Gene Body
Promoters
Enhancers
Mean Methylation
WT
R878H
3A
3L
EV
Dnmt3a R878H/+ Addback

fig. S7. Effects of retroviral addback *in vivo* in R878H bone marrow cells.

(**A**) Extent of correction of methylation values for all the R878H DMRs in non-transduced or transduced R878H samples. The vertical line drawn at 1.0 indicates remethylation to wild-type levels. (**B**) Extent of remethylation of R878H DMRs in *DNMT3A1* transduced *Dnmt3a*$^{R878H/+}$ bone marrow cells vs. *DNMT3L* transduced R878H bone marrow cells. (**C**) Mean CpG methylation levels from WGBS of bone marrow cells from WT, R878H, and transduced R878H samples. Mean values for all CpGs and designated regions of the genome are shown.

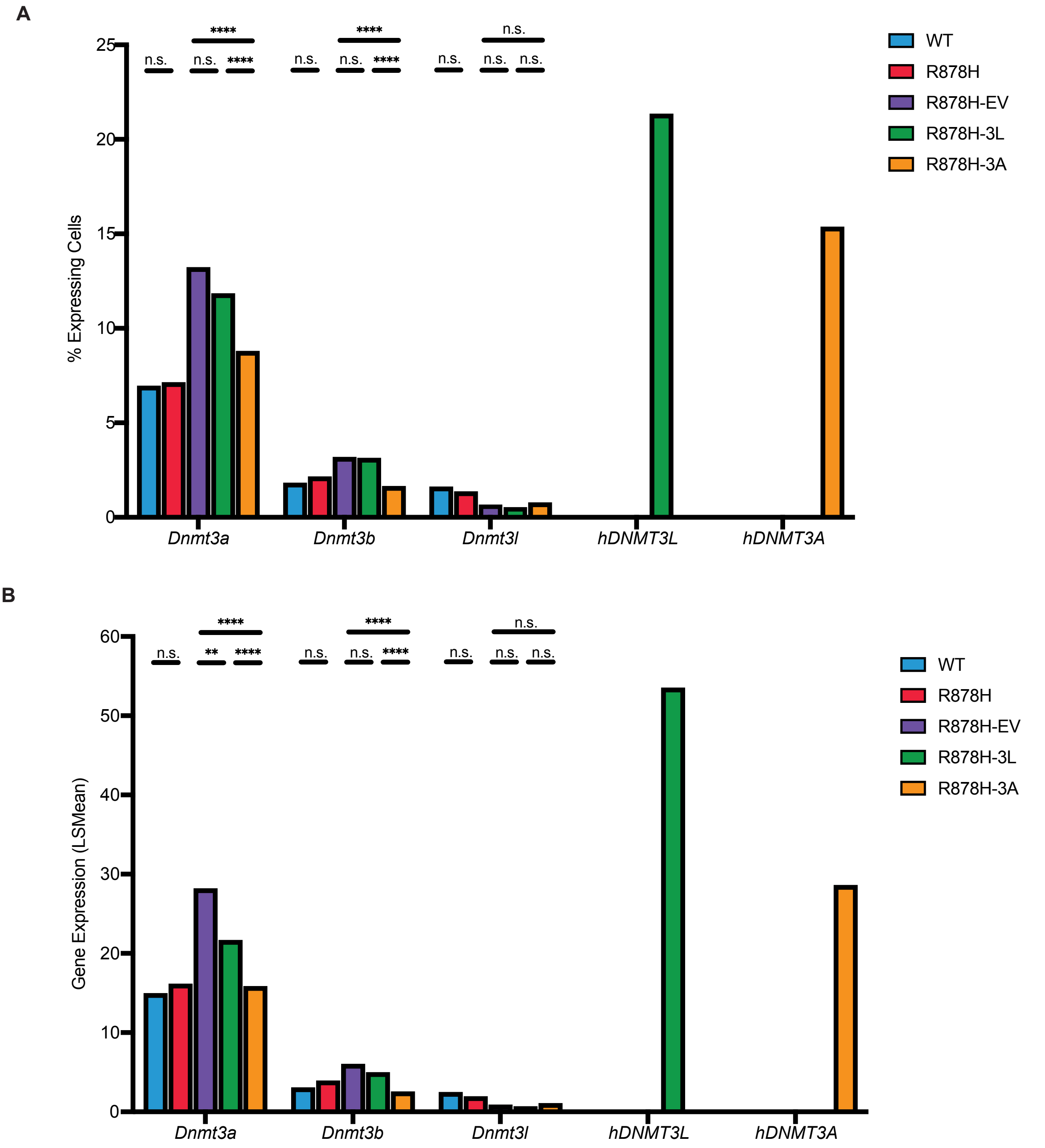
A
% Expressing Cells
0
5
10
15
20
25
n.s.
n.s.
****
****
n.s.
n.s.
****
****
n.s.
n.s.
n.s.
n.s.
Dnmt3a
Dnmt3b
Dnmt3l
hDNMT3L
hDNMT3A
WT
R878H
R878H-EV
R878H-3L
R878H-3A
B
Gene Expression (LSMean)
0
10
20
30
40
50
60
n.s.
**
****
****
n.s.
n.s.
****
****
n.s.
n.s.
n.s.
n.s.
Dnmt3a
Dnmt3b
Dnmt3l
hDNMT3L
hDNMT3A
WT
R878H
R878H-EV
R878H-3L
R878H-3A

fig. S8. Endogenous and exogenous expression levels of *Dnmt3a*, *Dnmt3b*, and *Dnmt3l,* in WT, *Dnmt3a*$^{R878/+}$, and addback bone marrow cells from scRNA-seq data. (**A**) Fraction of bone marrow cells expressing endogenous mouse *Dnmt3a*, *Dnmt3b*, or *Dnmt3l*, vs. exogenous, retrovirally expressed human *DNMT3A* and *DNMT3L* in the same samples. Addback data was obtained from the scRNA-seq data shown in Fig. 8, obtained one month after retroviral transduction and engraftment in mice. (**B**) Relative gene expression values in expressing cells for the same genes in the same samples.

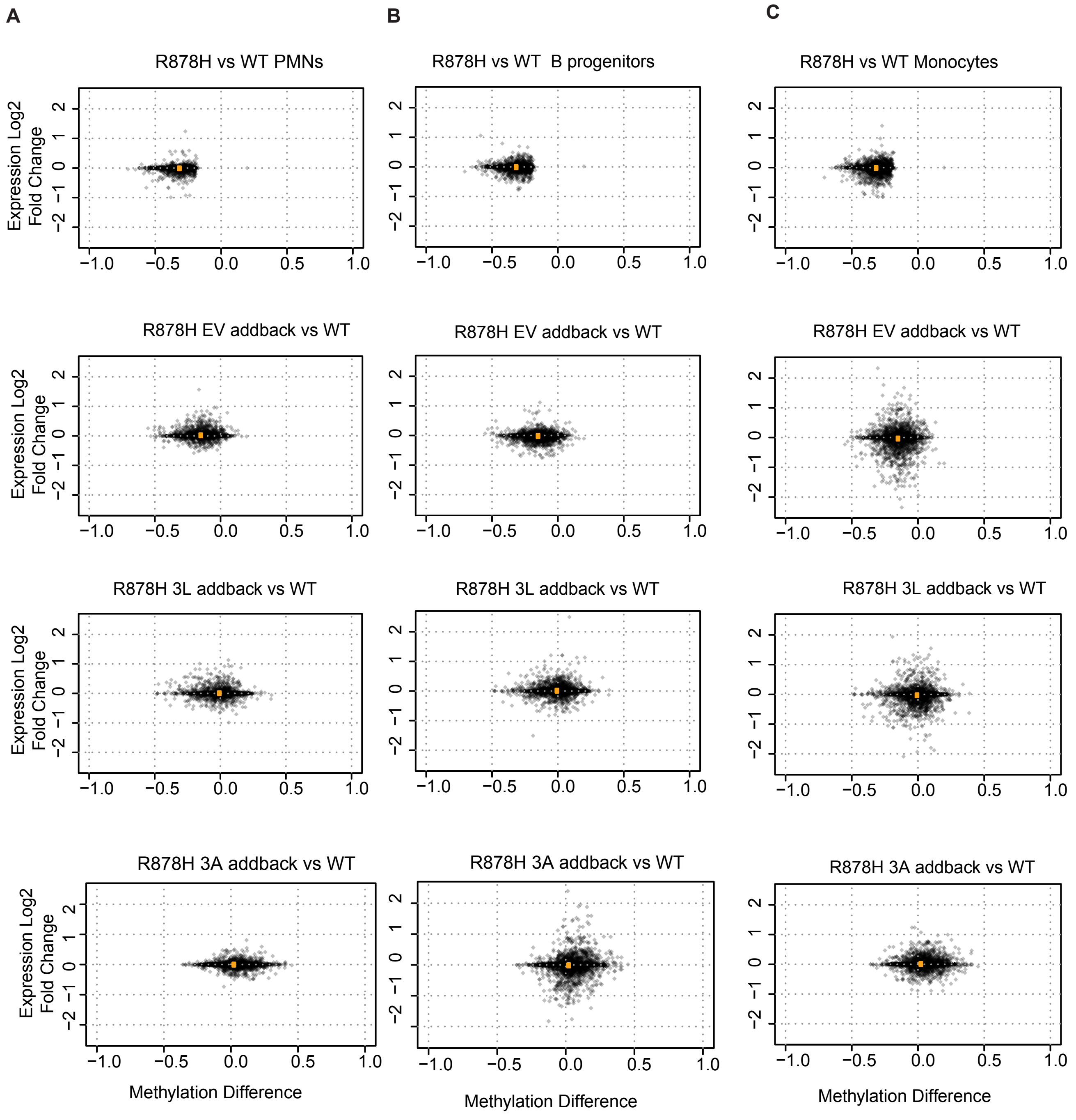
A
B
C
R878H vs WT PMNs
R878H vs WT B progenitors
R878H vs WT Monocytes
R878H EV addback vs WT
R878H EV addback vs WT
R878H EV addback vs WT
R878H 3L addback vs WT
R878H 3L addback vs WT
R878H 3L addback vs WT
R878H 3A addback vs WT
R878H 3A addback vs WT
R878H 3A addback vs WT
Expression Log2 Fold Change
Methylation Difference

fig. S9. DNA methylation and gene expression in R878H *in vivo* addback BM samples. (A, to C) Top panel in each: correlation between methylation and expression differences for individual genes within 10 Kb of a DMR defined by the comparison of R878H and WT bone marrow. Panels below represent the differences between WT values at these DMRs, and the addback samples for EV, DNMT3L, or DNMT3A. X axes: methylation differences at DMRs, showing the difference between WT and addback values for each DMR. Y axes: expression differences for genes within 10 Kb of these DMRs, comparing the values in WT cells and addback cells. (A) methylation vs. expression differences in PMNs, (B) methylation vs. expression differences in B cell progenitors, and (C) methylation vs. expression differences in monocytes.

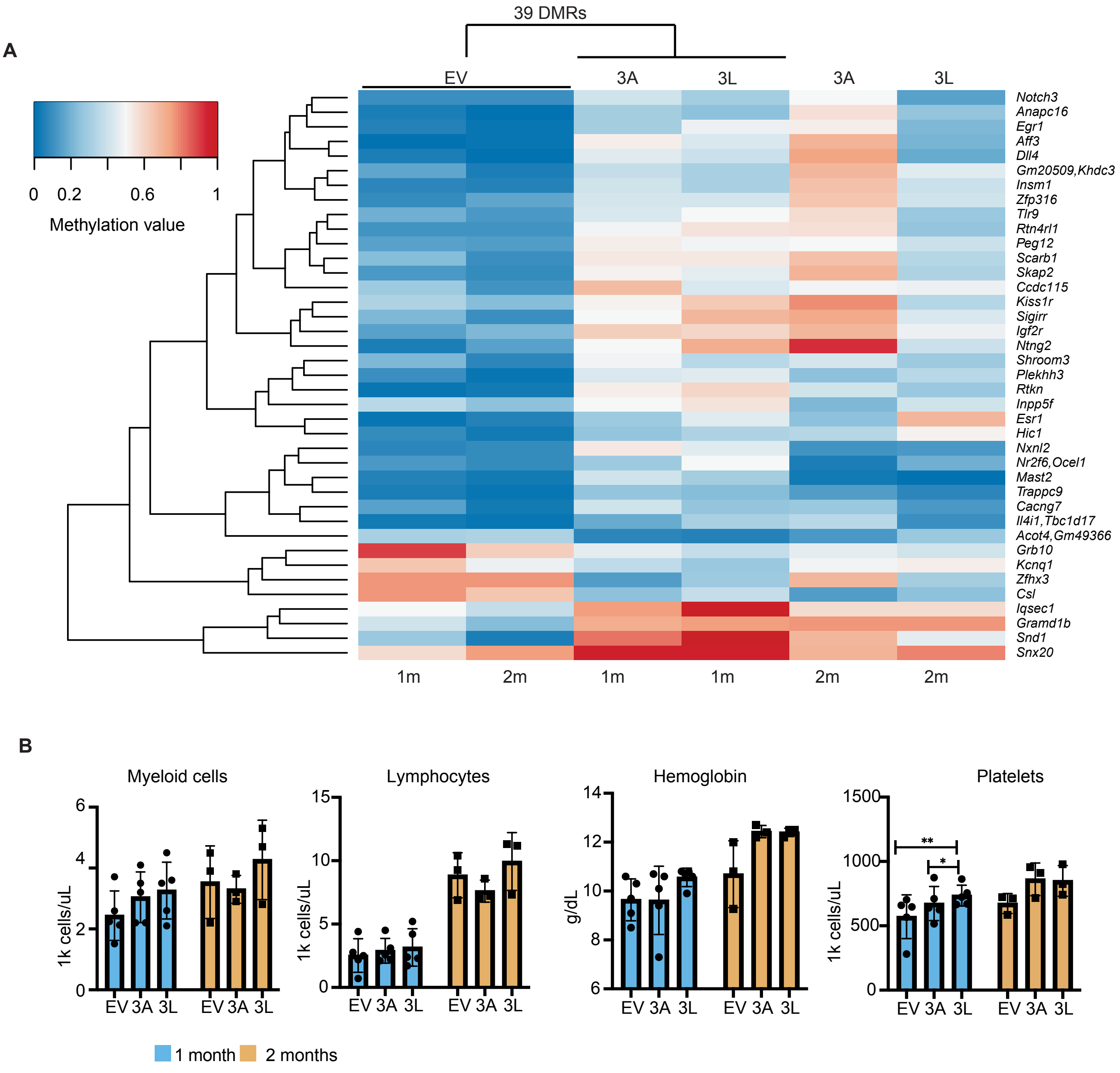
A
39 DMRs
EV
3A
3L
3A
3L
0
0.2
0.6
1
Methylation value
Notch3
Anapc16
Egr1
Aff3
Dll4
Gm20509,Khdc3
Insm1
Zfp316
Tlr9
Rtn4rl1
Peg12
Scarb1
Skap2
Ccdc115
Kiss1r
Sigirr
Igf2r
Ntng2
Shroom3
Plekhh3
Rtkn
Inpp5f
Esr1
Hic1
Nxnl2
Nr2f6,Ocel1
Mast2
Trappc9
Cacng7
Il4i1,Tbc1d17
Acot4,Gm49366
Grb10
Kcnq1
Zfhx3
Csl
Iqsec1
Gramd1b
Snd1
Snx20
1m
2m
1m
1m
2m
2m
B
Myeloid cells
Lymphocytes
Hemoglobin
Platelets
1k cells/uL
g/dL
EV 3A 3L EV 3A 3L
1 month
2 months

fig. S10. Effects of retroviral addback on DNA methylation in WT bone marrow cells *in vivo.* (**A**) Heatmap showing the methylation values for 39 DMRs defined by comparing pooled 1 month and 2-month EV-GFP addback samples, to 1-month DNMT3A1-GFP or DNMT3L-GFP retroviral addback samples transduced into WT bone marrow cells. DNA methylation values for the same DMRs were passively plotted for the 3A and 3L addback samples harvested 2 months after transduction. WGBS was performed on the GFP+ cells purified from the bone marrow samples from each vector at each time point. "EV" = empty vector. “3A” = *DNMT3A1* cDNA vector. "3L" = *DNMT3L* cDNA vector. (**B**) Data from the complete blood counts of transplanted mice from the same *in vivo* addback experiment. Myeloid cells include neutrophils, monocytes, eosinophils, and basophils. * indicates $p < 0.05$, and ** indicates $p < 0.01$, determined by an ANOVA test.

Caption sentences for supplementary Table S1 to S7

Table S1. Differentially methylated regions in *Dnmt3a* knockout mouse whole bone marrow cells compared to WT mouse bone marrow cells.

Table S2. Differentially methylated regions in *Dnmt3b* knockout mouse whole bone marrow cells compared to WT mouse bone marrow cells.

Table S3. Differentially methylated regions in *Dnmt3a* x *Dnmt3b* double knockout mouse whole bone marrow cells compared to WT mouse bone marrow cells.

Table S4. Differentially methylated regions in *Dnmt3a*$^{R878H/+}$ mouse whole bone marrow cells compared to WT mouse bone marrow cells.

Table S5. Unified differentially methylated regions from *Dnmt3a* knockout, *Dnmt3b* knockout, *Dnmt3a* x *Dnmt3b* double knockout, and *Dnmt3a*$^{R878H/+}$ mouse bone marrow cells compared separately with WT mouse bone marrow cells.

Table S6. Mass Spectrometry data for purified DNMT3A proteins.

Table S7. Differentially methylated regions in WT mouse bone marrow addback samples.
